# Arabidopsis PRC1 core component AtRING1 regulates stem cell-determining carpel development mainly through repression of class I *KNOX* genes

**DOI:** 10.1186/s12915-016-0336-4

**Published:** 2016-12-22

**Authors:** Donghong Chen, Anne M. Molitor, Lin Xu, Wen-Hui Shen

**Affiliations:** 1 Institut de Biologie Moléculaire des Plantes (IBMP), UPR2357 du CNRS, Université de Strasbourg, 12 rue du Général ZIMMER, 67084 Strasbourg, France; 2 College of Bioscience and Biotechnology, International Associated Laboratory of CNRS-Fudan-HUNAU on Plant Epigenome Research, Hunan Agricultural University, Changsha, 410128 China; 3 National Key Laboratory of Plant Molecular Genetics, CAS Center for Excellence in Molecular Plant Sciences, Institute of Plant Physiology and Ecology, Shanghai Institutes for Biological Sciences, Chinese Academy of Sciences, 300 Fenglin Road, Shanghai, 200032 China; 4 Present address: Institut de Genetique et de Biologie Moleculaire et Cellulaire, 1 rue Laurent Fries, 67404 Illkirch, France

**Keywords:** Polycomb, AtRING1, KNOX-I, Floral stem cell, Carpel development

## Abstract

**Background:**

Polycomb repressive complex 2 (PRC2)-catalyzed H3K27me3 marks are tightly associated with the *WUS*-*AG* negative feedback loop to terminate floral stem cell fate to promote carpel development, but the roles of Polycomb repressive complex 1 (PRC1) in this event remain largely uncharacterized.

**Results:**

Here we show conspicuous variability in the morphology and number of carpels among individual flowers in the absence of the PRC1 core components AtRING1a and AtRING1b, which contrasts with the wild-type floral meristem consumed by uniform carpel production in *Arabidopsis thaliana*. Promoter-driven GUS reporter analysis showed that *AtRING1a* and *AtRING1b* display a largely similar expression pattern, except in the case of the exclusively maternal-preferred expression of *AtRING1b*, but not *AtRING1a*, in the endosperm. Indeterminate carpel development in the *atring1a;atring1b* double mutant is due to replum/ovule-to-carpel conversion in association with ectopic expression of class I *KNOX* (*KNOX-I*) genes. Moreover, *AtRING1a* and *AtRING1b* also play a critical role in ovule development, mainly through promoting the degeneration of non-functional megaspores and proper integument formation. Genetic interaction analysis indicates that the AtRING1a/b-regulated *KNOX-I* pathway acts largely in a complementary manner with the *WUS-AG* pathway in controlling floral stem cell maintenance and proper carpel development.

**Conclusions:**

Our study uncovers a novel mechanistic pathway through which *AtRING1a* and *AtRING1b* repress *KNOX-I* expression to terminate floral stem cell activities and establish carpel cell fate identities.

**Electronic supplementary material:**

The online version of this article (doi:10.1186/s12915-016-0336-4) contains supplementary material, which is available to authorized users.

## Background

The development of both animals and plants relies on stem cells, which are defined by their ability to renew themselves and give rise to daughter cells that differentiate and contribute to tissue and organ formation. In higher plants, stem cells reside in meristems, and cell lineage is easily traceable due to the immobility of cells. The shoot apical meristem (SAM) initiates at the embryo stage, and continuously produces the aerial part of the plant during post-embryonic growth. Upon transition to the reproductive phase, SAM usually shifts to the fate of inflorescence meristem (IM) and subsequently generates floral meristems (FMs) from the IM flanks [1–4]. Distinct from the indeterminacy of SAM and IM, the determinate FM produces a fixed number of peripheral floral organs around a central population of stem cells that are consumed in the formation of carpels. The ovules emerge from the meristematic placenta within the carpel, undergo the production of the embryo sac (ES, the female gametophyte/megagametophyte), and upon double fertilization ultimately give rise to seeds [5, 6]. For female gametophyte development, firstly, a single and enlarged megasporocyte (also called megaspore mother cell, MMC) differentiates from the archesporial cell at the tip of the ovule primordium and undergoes meiosis to develop a tetrad of four haploid megaspores (developmental stage FG1). Normally the chalazal-proximal one survives and becomes the functional megaspore. This megaspore undergoes three rounds of mitotic division and cellularization to give rise to an eight-nucleate/seven-celled female gametophyte, which comprises three antipodal cells, two synergids, one central cell containing two unfused polar nuclei, and one egg cell (developmental stage FG5) [7].

The class I *KNOX* (*KNOX-I*) family gene *SHOOT MERISTEMLESS* (*STM*) and the feedback loop formed by *CLAVATA* (*CLV*) and *WUSCHEL* (*WUS*) have independent but complementary functions in shoot stem cell maintenance. For instance, *STM* prevents stem cell differentiation, while *WUS* specifies stem cell identity (reviewed in [8]). The knockdown mutants *stm* and *wus* display very similar flower phenotypes, such as the absence of carpels and a reduced number of other floral organs. In addition to *STM*, the other *KNOX-I* family genes *KNAT1*/*BREVIPEDICELLUS* (*BP*), *KNAT2*, and *KNAT6* may also have a role in carpel development because overexpression of either *STM* or *KNAT2* can induce ectopic carpel formation and ovule-to-carpel homeotic conversion within the gynoecium [9]. Very importantly, *AGAMOUS* (*AG*) plays a key role in the termination of floral stem cell maintenance. At flower developmental stage 3, WUS together with LEAFY (LFY) activate *AG*, which in turn shuts off *WUS* expression at stage 6, leading to the termination of stem cell maintenance and the initiation of carpel primordia [10–14]. Either *ag*, displaying spatially restricted but delayed *WUS* extinction, or *clv*, displaying an enlarged *WUS* expression domain, is sufficient to induce FM indeterminacy [13–18]. Thus, *AG* combined with the *CLV*-*WUS* feedback loop regulates carpel development, conveniently named the *WUS*-*AG* pathway. Recent studies demonstrate that some Polycomb group (PcG) proteins play an essential role within the *WUS*-*AG* pathway to terminate floral stem cell fate [19, 20].

PcG proteins constitute two major types of complexes: Polycomb repressive complex 2 (PRC2), which catalyzes histone H3 lysine 27 trimethylation (H3K27me3) on target chromatin, and PRC1, which acts as both the H3K27me3 reader and the histone H2A lysine 119 monoubiquitination (H2AK119ub1) writer. Arabidopsis PRC2 components are able to form at least three different complexes involved in somatic cell fate determinacy, vegetative development maintenance, vernalization, flower timing regulation, and seed development (reviewed in [21]). Arabidopsis PRC1 core components, including LIKE HETEROCHROMOTIN PROTEIN1 (LHP1), AtBMI1, and AtRING1, display different evolutionary conservation [22]. Though LHP1 can interact with AtRING1 and AtBMI1 in vitro [23], the mutant phenotype of *lhp1* shows some degree of difference from that of the *atring1a;atring1b* or *atbmi1a;atbmi1b* double mutant. Furthermore, LHP1 was recently reported to co-purify with the PRC2 complex in vivo [24, 25], indicating that LHP1 is more closely associated with PRC2 in this specific context than PRC1. Arabidopsis PRC1 RING finger proteins AtRING1 and AtBMI1 act as the most conserved components involved in preventing seed germination and development of somatic embryo traits [23, 26, 27], maintaining stem cell identity [28], and promoting floral transition [29]. Intriguingly, *atring1a;atring1b* mutants display abnormal flower developmental phenotypes, yet the underlying mechanisms remain to be investigated.

In this study, we show that *AtRING1a* and *AtRING1b* play an essential role in Arabidopsis floral stem cell maintenance and carpel development, primarily via repression of the *KNOX-I* family genes. Both *AtRING1a* and *AtRING1b* genes display very similar expression patterns throughout the whole plant life cycle, except for the imprinting expression of *AtRING1b*, but not *AtRING1a*, in the endosperm. Indeterminate carpel growth in the *atring1a;atring1b* mutant is associated with homeotic replum-to-carpel and ovule-to-carpel conversions. Further molecular and genetic analyses demonstrate that *AtRING1a/b* modulate floral stem cell activity and carpel development, mainly through repression of the *KNOX-I* pathway. Lastly, our analyses indicate that defective ovule development in the *atring1a;atring1b* mutant is essentially due to survival of non-functional megaspores, growth arrest of integuments, and overproliferation of the nucellus.

## Results

### *AtRING1a* and *AtRING1b* display overlapping as well as different tissue-specific expression patterns

**Fig. 1:**
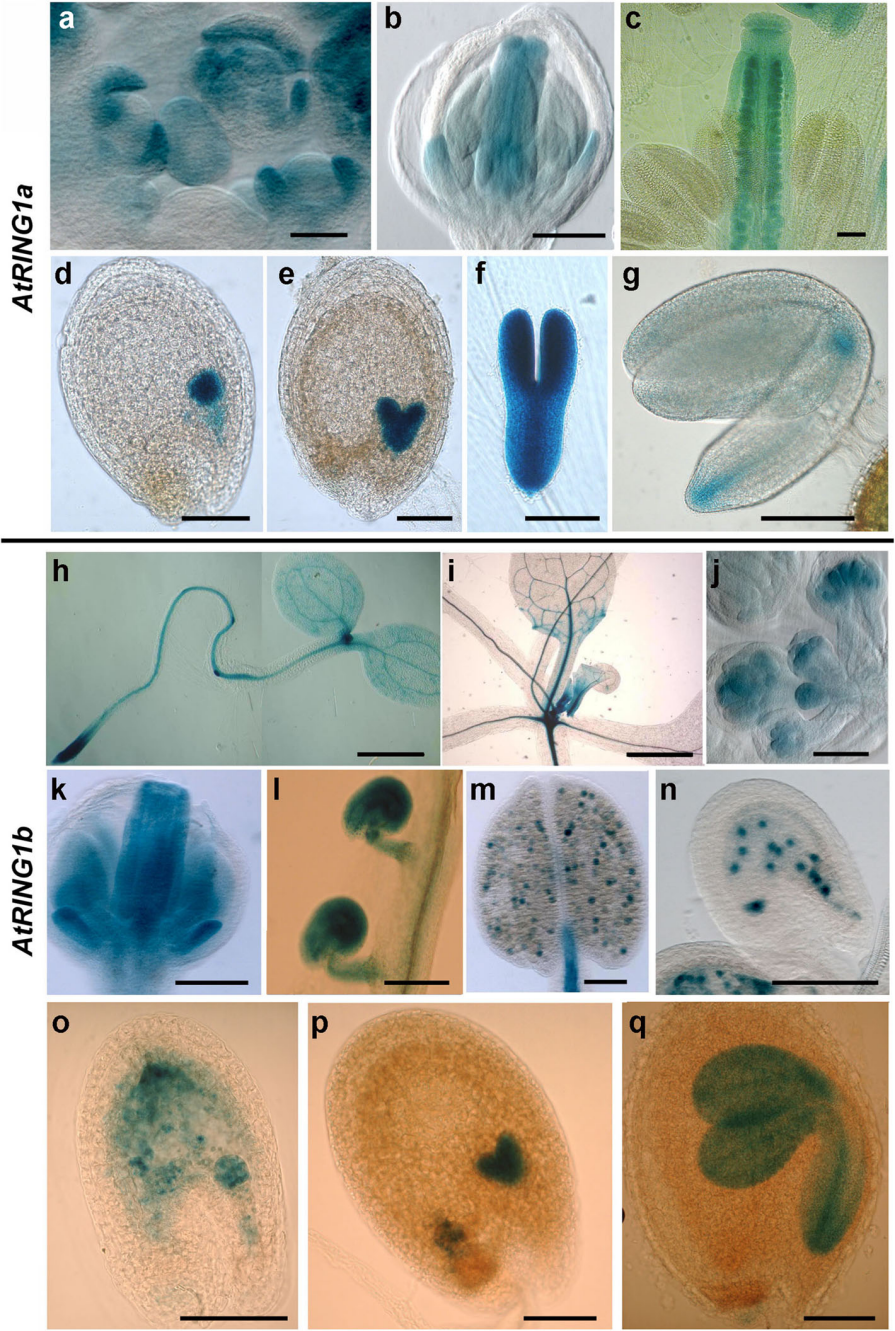
*AtRING1a* and *AtRING1b* exhibit similarities yet some differences in expression pattern at the reproductive stage. **a**–**g** Expression pattern of AtRING1a in *AtRING1a*::*AtRING1a*-*GUS* transgenic lines. **a** Inflorescence. Note strong GUS staining in sepal primordia. **b** Floral bud at stages 8 and 9 of flower development. Note strong GUS staining in early floral organs. Unless otherwise indicated, flower developmental stages are defined according to [60]. **c** Emerging flower. Note strong GUS staining in ovules, but none in mature pollen. **d** Globular stage of embryo development. **e** Heart stage of embryo development. **f** Linear cotyledon stage of embryo development. **g** Mature green stage of embryo development. **h**–**q** Expression pattern of AtRING1b detected in *AtRING1b*::*AtRING1b*-*GUS* transgenic lines. **h** Three-day-old seedling. **i** One-month-old seedling. **j** Inflorescence. **k** Flower bud at stage 8. **l** Mature ovule. **m** Mature pollen and filament. **n** Fertilized ovule at 2 days after pollination (*DAP*). **o** Globular stage of embryo development. **p** Heart stage. **q** Bending cotyledon stage. Bars = 100 μm, except 50 μm in **a**, **b**, **j**, and **k**; 1 mm in **h** and **i**

Reverse transcription polymerase chain reaction (RT-PCR) detected broad expression of *AtRING1a* and *AtRING1b* in multiple types of plant organs [28]. A more detailed analysis using the *AtRING1a*::*AtRING1a*-*GUS* reporter line showed that *AtRING1a* is expressed in the SAM, root apical meristem (RAM), the junction between shoot and root, and young leaves [23]. Here we extend the *AtRING1a*::*AtRING1a*-*GUS* expression analysis in different reproductive floral organs as well as during embryogenesis. GUS staining showed that *AtRING1a* is strongly expressed in floral organ primordia of inflorescences (Fig. 1a), in gynoecia and ovules (Fig. 1b and c), and in embryos throughout diverse developmental stages (Fig. 1d–g). We further verified *AtRING1a* expression pattern by in situ hybridization analysis of endogenous gene transcripts in wild-type (WT) plants (Additional file 1: Figure S1). The data confirmed the expression patterns determined by GUS reporter analysis and also showed that *AtRING1a* transcripts are detectable in both microsporocytes and megasporocytes (Additional file 1: Figure S1E and G).

To study the tissue specificity of *AtRING1b* expression, we constructed an *AtRING1b*::*AtRING1b*-*GUS* reporter containing *AtRING1b* full-length genomic DNA and a ~1.6-kb promoter upstream of the translation start site. GUS staining was performed using three independent transgenic lines; similar expression patterns were observed across all lines. During vegetative development, expression of *AtRING1b*::*AtRING1b*-*GUS* was detected at high levels in SAM and RAM (Fig. 1h) and at moderate levels in young leaves and vasculature (Fig. 1i). During reproductive development, the GUS signal was strong in inflorescence floral organ primordia (Fig. 1j), gynoecia and ovules (Fig. 1k and l), pollen grains (Fig. 1m), early endosperm (Fig. 1n–p), and in embryos throughout a variety of developmental stages (Fig. 1o–q). These data show that *AtRING1a* and *AtRING1b* have largely overlapping tissue-specific expression patterns. Yet, differences also exist between *AtRING1a* and *AtRING1b*. Most remarkably, strong expression of *AtRING1b* was detected in mature pollen grains (Fig. 1m), whereas *AtRING1a* expression was barely detectable (Fig. 1c). Likewise, strong expression of *AtRING1b* was detected in the endosperm during early seed development until the globular embryo stage (Fig. 1n–p), whereas *AtRING1a* expression was undetectable (Fig. 1d).

### *AtRING1b* expression in endosperm is maternally imprinted

**Fig. 2:**
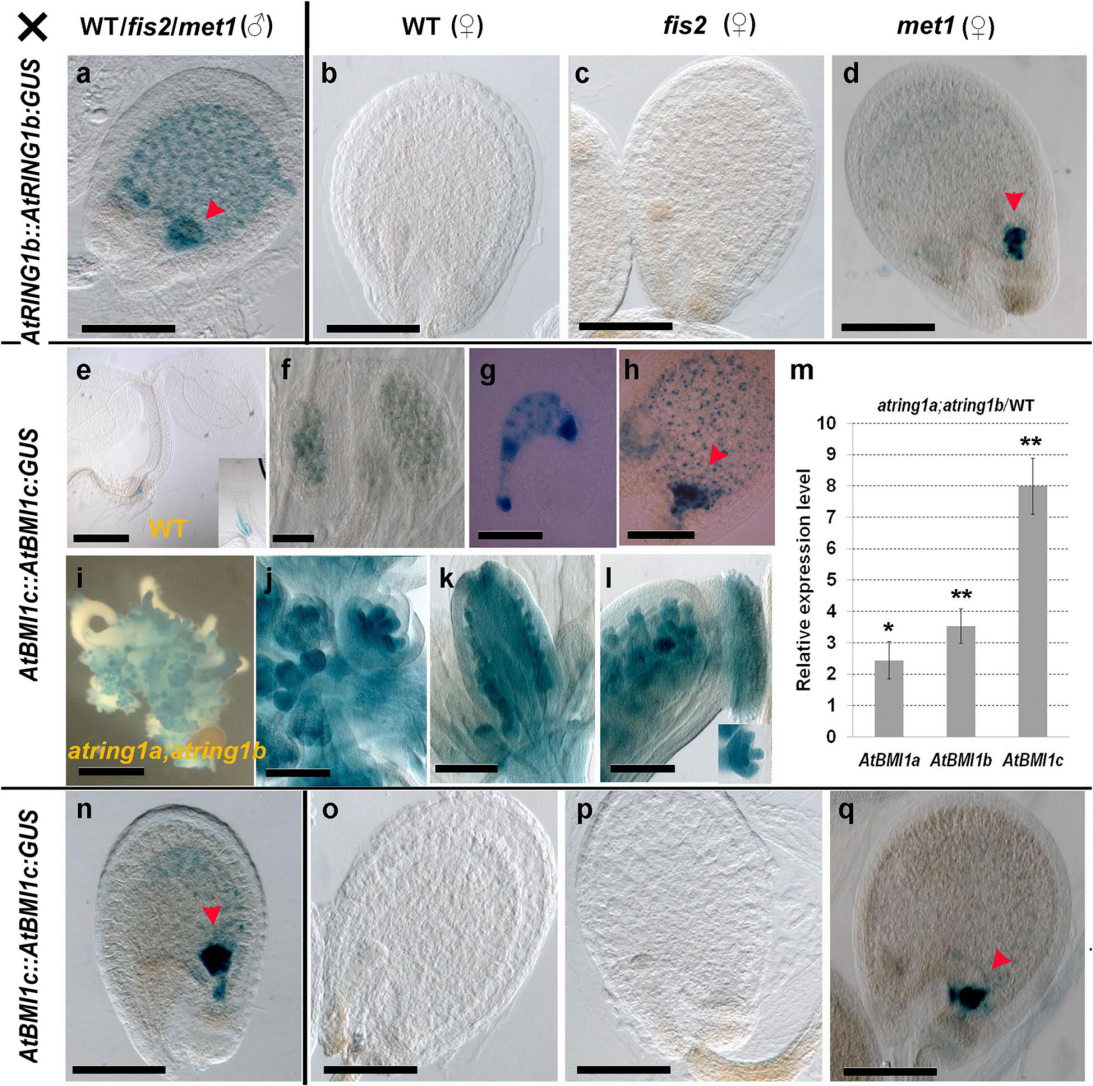
*AtRING1b* displays maternally imprinted expression in endosperm. **a**–**d** *AtRING1b* expression was analyzed in the seeds after reciprocal crosses of *AtRING1b*::*AtRING1b*-*GUS* (Columbia, Col background) with Col, *fis2*, or *met1-3* at 3 DAP. **a** *AtRING1b*::*AtRING1b*-*GUS* (♀) × Col (♂). Similar patterns were observed for *AtRING1b*::*AtRING1b*-*GUS* (♀) × *fis2* (♂) and *AtRING1b*::*AtRING1b*-*GUS* (♀) × *met1-3*. (**b**) Col (♀) × *AtRING1b*::*AtRING1b*-*GUS* (♂). **c** *fis2* (♀) × *AtRING1b*::*AtRING1b*-*GUS* (♂). **d** *met1-3* (♀) × *AtRING1b*::*AtRING1b*-*GUS* (♂). **e**–**h** Expression pattern of *AtBMI1c* investigated by analysis of *AtBMI1c*::*AtBMI1c*-*GUS* transgenic lines. **e** Staining in RAM (*inset*) and junction between root and shoot of 1-week-old seedling. **f** Developing anther. **g** Fertilized ovule at 1 DAP. **h** Developing seed at globular stage. **i**–**l** Expression pattern of *AtBMI1c* in *atring1a;atring1b* mutant in *AtBMI1c*::*AtBMI1c*-*GUS* lines. **i** Embryo-like structure produced in 2-week-old *atring1a;atring1b* seedling. **j** Inflorescence. **k** Developing gynoecium (about stage 9). **l** Developing gynoecium (about stage 12) and young ovule (*inset*). **m** Increased levels of *BMI1* transcripts in the *atring1a;atring1b* mutant detected by qRT-PCR (Student’s *t* test, *p < 0.05, **p < 0.01). Error bars represent SD for three biological replicates. **n**–**q** *AtBMI1c* expression analyzed in the *AtBMI1c*::*AtBMI1c*-*GUS* construct in seeds after reciprocal crosses, as described in **a**–**d** for *AtRING1b*::*AtRING1b*-*GUS. Arrowheads* indicate chalazal endosperm. Bars = 100 μm, except 500 μm in **e** and **i**, and 50 μm in **f**

The intriguing endosperm expression pattern of *AtRING1b*::*AtRING1b*-*GUS* prompted us to test whether *AtRING1b* is a parentally imprinted gene. Based on reciprocal crosses, we found that GUS activity was detected in the endosperm when *AtRING1b*::*AtRING1b*-*GUS* ovules were fertilized by WT pollen (Fig. 2a). In contrast, GUS activity was undetectable in the endosperm when WT ovules were fertilized by *AtRING1b*::*AtRING1b*-*GUS* pollen (Fig. 2b). This result indicates that only the maternal, but not the paternal, *AtRING1b* allele is actively expressed in endosperm cells. To investigate whether PcG silencing and/or DNA methylation is involved in parental imprinting of *AtRING1b*, we performed reciprocal crosses using the PRC2 mutant *fertilization independent seed 2* (*fis2*) and the *DNA METHYLTRANSFERASE1* mutant *met1-3* [30, 31]. The *fis2* mutant behaved similarly to WT and displayed GUS activity only when *AtRING1b*::*AtRING1b*-*GUS* was maternally derived but not paternally derived (Fig. 2c). In contrast, *met1-3* displayed a different pattern, since GUS activity was detected in endosperm cells when *AtRING1b*::*AtRING1b*-*GUS* was either maternally or paternally derived (Fig. 2d).

The *AtBMI1c* gene was identified as maternally expressed in previous studies based on single nucleotide polymorphism (SNP) and RT-PCR analyses of seeds derived from crosses of different Arabidopsis ecotypes [32, 33]. In this study, we generated and tested expression of an *AtBMI1c*::*AtBMI1c*-*GUS* reporter construct containing *AtBMI1c* full-length genomic DNA and its ~2.1-kb upstream promoter region. Weak expression of *AtBMI1c*::*AtBMI1c*-*GUS* was detected in the root tip, at the junction between shoot and root, and in pollen grains (Fig. 2e and f). High levels of expression were observed in the embryo sac and endosperm (Fig. 2g and h). Consistent with previous RT-PCR data [23], expression of *AtBMI1c*::*AtBMI1c*-*GUS* was drastically increased in several tissue types in the *atring1a;atring1b* mutant (Fig. 2i–m). Based on reciprocal genetic crosses, we found that *AtBMI1c*::*AtBMI1c*-*GUS* has an imprinted expression pattern (Fig. 2n–q) similar to that of *AtRING1b*::*AtRING1b*-*GUS* (Fig. 2a–d). Thus, both *AtRING1b* and *AtBMI1c* show maternally imprinted expression in the endosperm, and their genomic imprinting is regulated by MET1-dependent CG DNA methylation but not by PRC2 silencing.

### Loss of *AtRING1* drastically affects gynoecium development

**Fig. 3:**
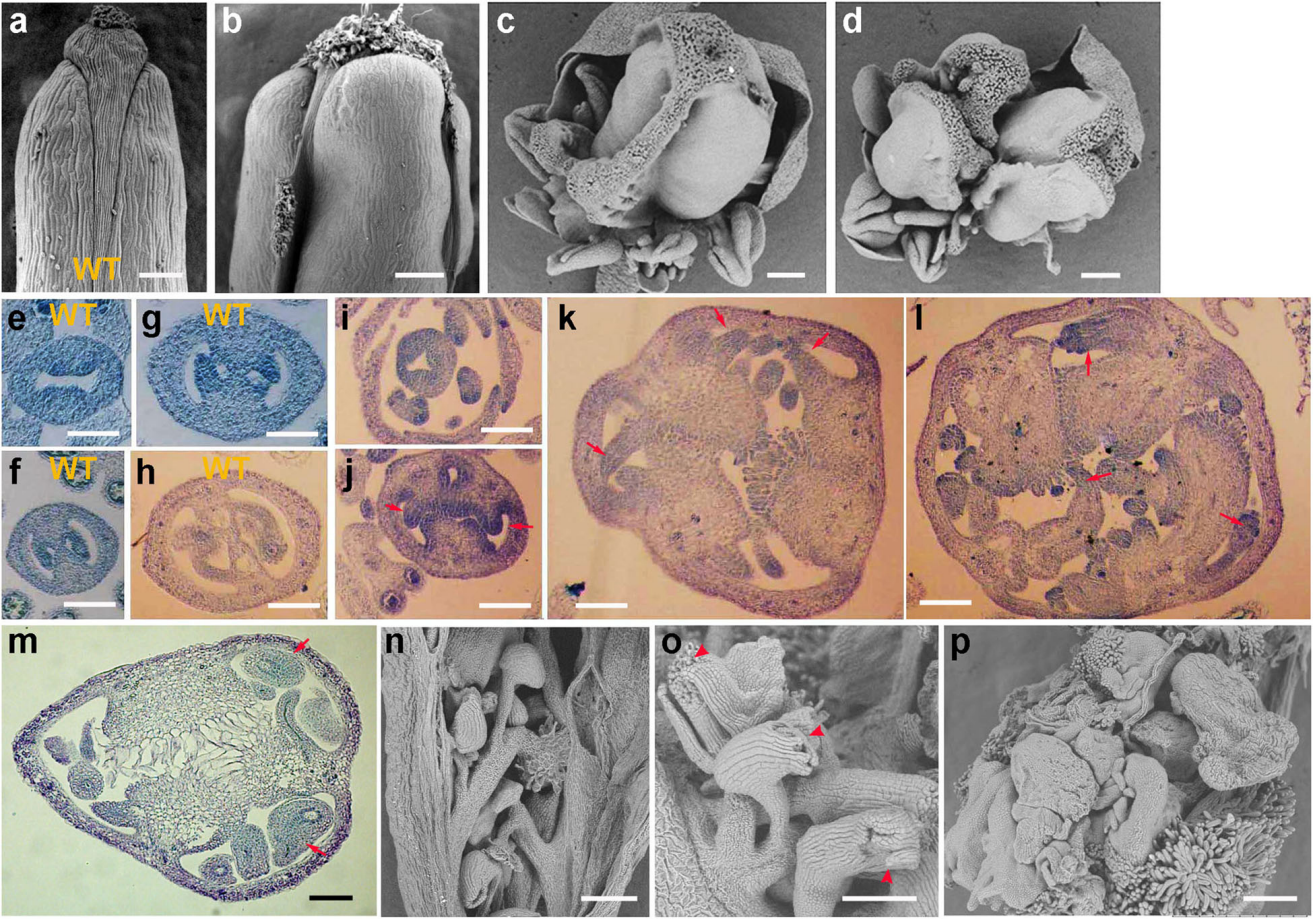
Indeterminate carpel development in *atring1a;atring1b* mutant. **a** Wild-type (WT) carpel. **b**–**d** Defective carpel development in *atring1a;atring1b* mutant. **b** Bulged carpels. **c** Stigma papilla-like structures growing outside the replums between carpels. **d** Unclosed carpel development. **e**–**h** Cross sections of various stages of carpel and ovule development in WT flower. **e** Ovule primordia initiation within the gynoecial cylinder of a stage 8 WT flower. **f** Gynoecial cylinder in a stage 9 WT flower. **g** Gynoecial cylinder in a stage 11 WT flower. **h** Mature ovule within a stage 13 WT flower. **i**–**m** Cross sections of various stages of carpel and ovule development in *atring1a;atring1b* mutant. Medial ridges fail to fuse but continue to expand and produce stigmatic papilla-like tissues on the top. *Arrows* denote various stages of ovules. **n**–**p** SEM observation of ectopic carpelloid features inside mutant mature gynoecia. **n** Ectopic additional ovules arising from overproliferated carpel-like structure inside the mutant central gynoecium. **o** Carpelloid-like ovules with stigma papilla-like organs (*arrowheads*) on the top. **p** Overproliferating carpel-like structures crowded inside a gynoecium. Bars =200 μm, except 10 μm in **e**–**l**

Compared with the WT Arabidopsis gynoecium consisting of two fused carpels (Fig. 3a), the *atring1a;atring1b* double mutant displays diversely modified gynoecium phenotypes ranging from bulged/supernumerary to unfused carpels (Fig. 3b–d) as revealed through scanning electron microscopy (SEM) examination. To obtain further insight into the cytological basis underlying the defective gynoecium development of the *atring1a;atring1b* double mutant, we created paraffin sections of gynoecium. Within WT gynoecia, marginal tissues of the carpel fusion give rise to two medial ridges (replums) that grow toward each other and eventually meet and fuse to form the septum (Fig. 3e–h). The meristematic replum is the region which gives rise to the placenta, ovules, and septa [34]. On the other hand, ovule primordia emerge from the placenta at both flanks of the replum. Then, ovule develops through MMC differentiation, functional megaspore production, and mature embryo sac formation processes. Within the *atring1a;atring1b* mutant pistil, most ovule primordia are seemingly initiated from the placental region of a high number-carpel fused gynoecium (Fig. 3i and j). But later on, the replum loses its determinacy of septum fate and constantly overproliferates, developing into carpel-like organs (Fig. 3k–m) which can still produce some ovules on the margins and stigma papilla-like structures at the apex (Fig. 3n). Sometimes, the replum develops carpelloid tissues outside the gynoecium (Fig. 3c), and stigmatic features can be observed on the top of some ovules (Fig. 3o and p), together showing an ectopic overproliferation of carpelloid tissues. Thus, we conclude that homeotic conversions of replum-to-carpel and ovule-to-carpel characterize the basis of defective carpel development in the *atring1a;atring1b* double mutant.

### Loss of AtRING1 function leads to defective ovule and embryo sac development

**Fig. 4:**
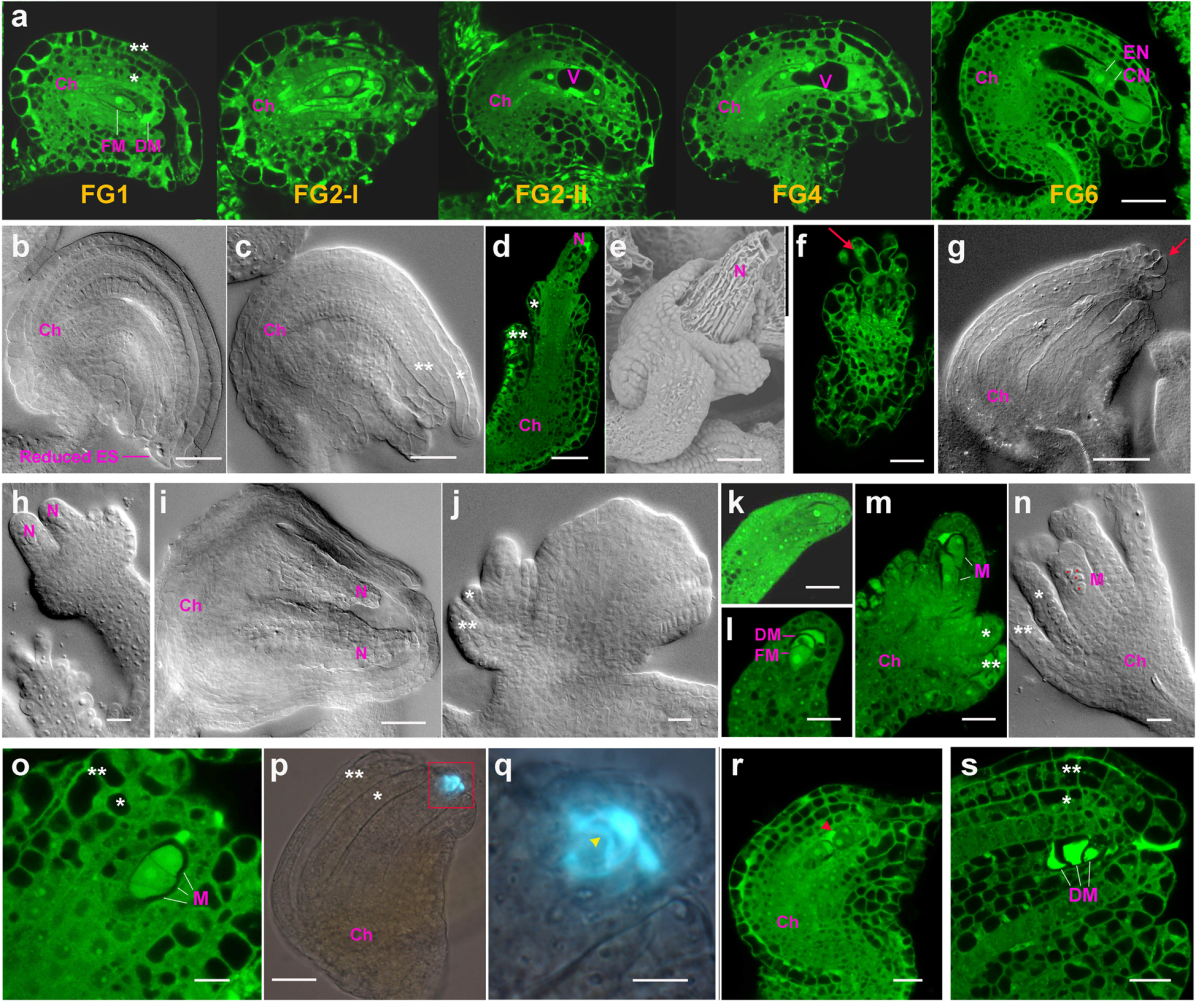
Phenotypic analysis of *atring1a;atring1b* during ovule and ES development. **a**. Ovule and ES development stages in WT. Functional (*FM*) and degenerated megaspores (*DM*) are showed at FG1 stage. Strong autofluorescence indicates DM. A two-nucleate ES is shown in early FG2 (FG2-I); an enlarged central vacuole and a small chalazal vacuole appear in the late FG2 (FG2-II). A four-nucleate ES develops at FG4. A mature seven-celled ES is produced at FG6. (ES stages are defined according to [7].) **b**–**s** Ovule and ES development in the *atring1a;atring1b* strong mutants. **b** Reduced ES and mildly proliferated nucellus in a mature ovule. **c** No obvious ES development. **d**, **e** Arrested outer integument and overproliferated nucellus. **f** Young mutant ovule with stigmatic papilla-like structure arising from nucellar epidermis. **g** Ovule-to-carpel conversion. **h**, **i** Double nucelli in one ovule. **j** Outer integument develops into leaf-like structure. **k** A normally differentiated MMC at stage FG0. **l** Developing ovule primordium at stage FG1 without integument initiation. **m** Developing ovule primordium with two surviving megaspores but severely inhibited integument growth. **n** All four surviving megaspores become arrested at later stage. **o** Three arrested megaspores at later stage. **p** Aniline blue staining showing growth arrest of two megaspores in a mature ovule. Callose accumulation indicated by bright cyan. **q** Close-up view of **p**. *Yellow* arrowhead indicates cell plate. **r** One of several megaspores can occasionally undergo one mitotic division to enter into FG2 stage (*arrowhead*). **s** Arrested megaspores gradually undergo degeneration during later development. * inner integument, ** outer integument, *Ch* chalazal, *CN* central cell nucleus, *DM* degenerated megaspore, *EN* egg cell nucleus, *FM* functional megaspore, *M* megaspore, *N* nucellus, *V* large vacuole. Bars = 10 μm, except 50 μm in **a**–**e**, **g**, **i**, and **p**

In WT Arabidopsis, the ovule exhibits determinate growth, ultimately developing into a seven-celled and eight-nucleus ES enclosed by inner and outer integuments. But in the *atring1a;atring1b* mutant, ovule development occasionally adapts carpel fate (Fig. 3o and p) and becomes indeterminate. In order to dissect abnormalities of ovule and ES development in the *atring1a;atring1b* mutant, confocal laser scanning microscopy (CLSM) and differential interference contrast (DIC) observations were carried out. WT ovules show characteristics typical of different developmental stages: FG1 to FG6 (Fig. 4a; [7]). Defective mutant ovules fall into two major classes: class I (~8%, *N* = 145) displaying reduced ES with relatively normal nuclear division and integument development (Fig. 4b), and class II (~92%, *N* = 145) displaying arrested or no ES with abnormal integument formation (Fig. 4c). Different extents of integument or nucellus defects are observed. In normal WT ovule development, prior to anthesis the nucellus degenerates, leaving the ES in direct contact with the endothelium (integumentary tapetum), differentiated from the inner layer of the inner integument (FG1 and FG2-I, Fig. 4a; [35, 36]). In contrast, most *atring1a;atring1b* mutant ovules display an overproliferated nucellus growing out of outer and inner integuments (Fig. 4d and e). Compared with the double integuments growing to cover and enclose the nucellus during normal ovule development, outer integument expansion in some mutant ovules (~30%, *N* = 145) is severely inhibited. In WT ovules the outer integument primordium initiates and grows asymmetrically, with only its adaxial side extending significantly. In contrast, uniform extension of the integuments surrounding the nucellus occurs in about 6% of the mutant ovules, resulting in symmetric integument growth lacking an S-shaped curvature. Furthermore, ~10% of the mutant ovules exhibit homeotic conversion of integument/nucellus-to-stigmatic papilla (Fig. 4f and g). In some ovules (~5%) two nucelli are enclosed within the same integuments (Fig. 4h and i). Occasionally, the outer integument in the mutant ovule is transformed into a blade-like structure (Fig. 4j), which is reminiscent of the supposed ancestral origin, a cupule with a leaf-like structure surrounding one or more ovules [37].

In spite of integument and nucellus growth defects, one MMC can differentiate within each nucellus of the *atring1a;atring1b* mutant ovule (Fig. 4k) as normally found in WT ovules. One MMC undergoes meiosis to produce four megaspores, from which three degenerate and one becomes a functional megaspore (FM, Fig. 4l). In most of the mutant ovules, megaspore degeneration is disturbed, leading to survival of more than one megaspore (Fig. 4m–o). Aniline blue staining [38] showed callose accumulation (Fig. 4p and q), suggesting that the surviving megaspores go through growth arrest in subsequent stages. Occasionally, one of the surviving megaspores can undergo one cycle of mitotic division, resulting in the coexistence of megaspores and FG2-stage ES with two nuclei (Fig. 4r). Nevertheless, these surviving megaspores or FG2-stage ES eventually undergo growth arrest and degenerate (Fig. 4s).

Taken together, our observations indicate that *AtRING1a* and *AtRING1b* regulate ES and ovule development through determination of ovule identity, promotion of accurate megaspore degeneration, and inhibition of nucellus overproliferation.

### AtRING1 is required for suppression of *KNOX*-*I* genes

**Fig. 5:**
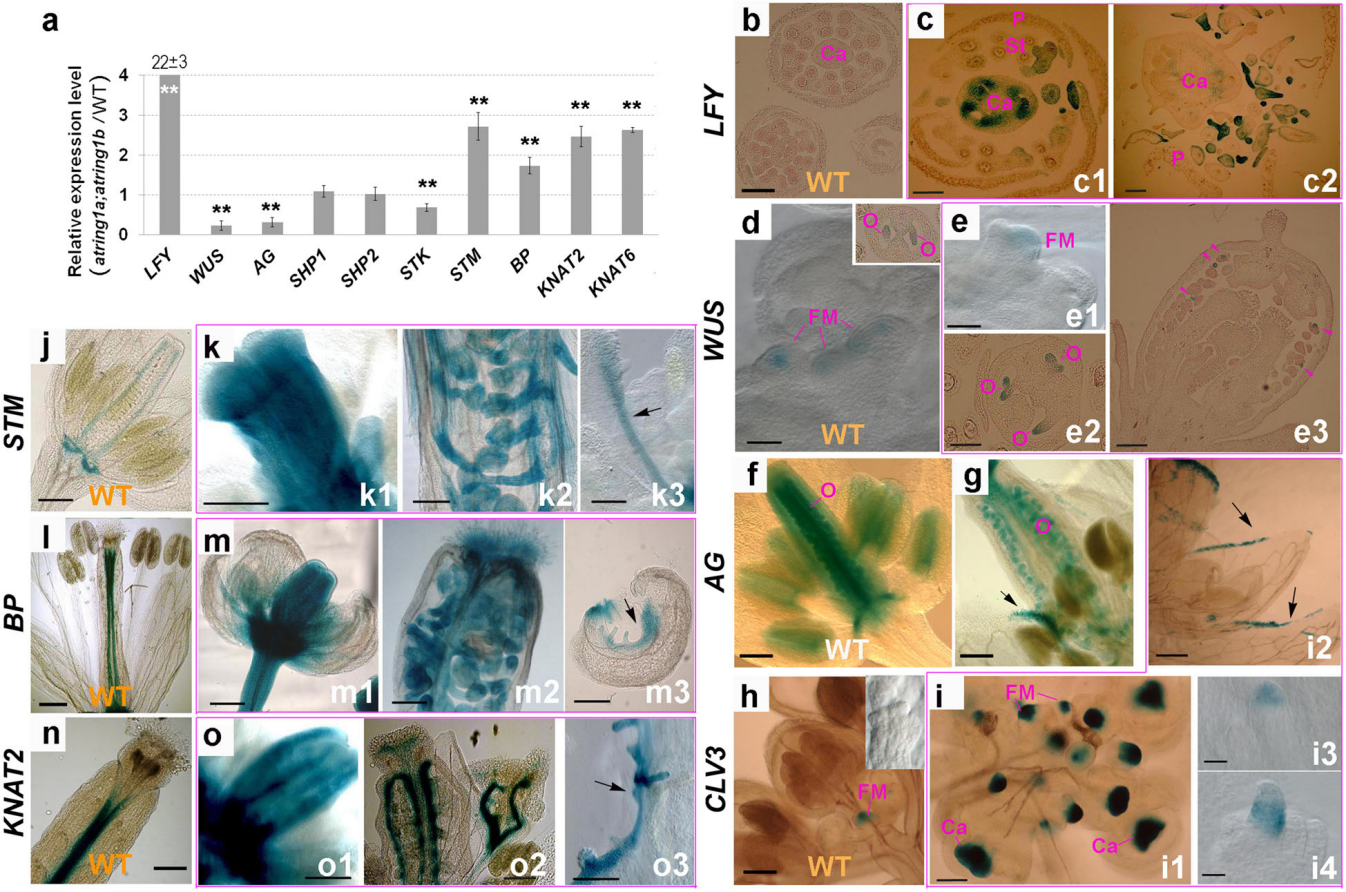
Expression pattern of *WUS*-*AG* and *KNOX*-*I* pathway-related genes in *atring1a;atring1b* mutant. **a** Expression profiles of select key genes of both stem-cell determining pathways and three *AG*-related genes in floral buds of *atring1a;atring1b* mutant detected using qRT-PCR (Student’s *t* test, **p < 0.01). Error bars represent SD from three biological replicates. **b**, **c** *LFY* expression pattern detected using *LFY*::*GUS* reporter. **b** No staining in WT developing carpel. **c** Strong staining in developing carpel (*c1*) and in extra floral organs between petal and carpel (*c2*) in *atring1a;atring1b* mutant. **d**, **e** *WUS* expression pattern detected using *WUS*::*GUS* reporter. **d** Staining in the WT floral meristem and in nucellus of developing ovule (*inset*). **e** Staining in floral meristem (*e1*) and in nucellus of developing primary (*e2*) and secondary ovules (*e3*) in *atring1a;atring1b* mutant. **f**, **g** *AG*::*GUS* reporter showing that WT (**f**) has stronger staining in developing carpel and anther compared to *atring1a;atring1b* mutant (**g**). *Arrow* indicates signal in ectopic stigma papillae in sepal. **h**, **i** *CLV3* expression pattern detected using *CLV3*::*GUS* reporter. **h** Staining in the WT floral meristem, but not in ovule primordium (*inset*). **i** Staining in FM and developing carpel (*i1*), in the ectopic papillae of carpelloid sepal (*i2*), and in nucellus of developing ovule (*i3* and *i4*) in *atring1a;atring1b* mutant. **j**, **k** *STM* expression detected using *STM*::*GUS* reporter. **j** GUS activity at the base and placenta of WT flower at stage 12. **k** Staining in the early carpel development (*k1*), mature ovule (*k2*), and ectopic papilla (*k3*) in *atring1a;atring1b* mutant. **l**, **m** *BP* expression detected using *BP*::*GUS* reporter. **l** Staining at the base and placenta of WT flower. **m** Staining in the early carpel (*m1*), mature ovule (*m2*), and ectopic carpel (*m3*) in *atring1a;atring1b* mutant. **n**, **o** *KNAT2* expression detected using *KNAT2*::*GUS* reporter. **n** Staining at the base and placenta of WT flower. **o** Staining in the early carpel (*o1*), in the placenta of mature carpel (*o2*), and in the ectopic carpel (*o3*) in *atring1a;atring1b* mutant. *Ca* carpel, *FM* floral meristem, *O* ovule, *P* petal, *St* stamen. Bar = 100 μm, except 50 μm in **b**–**e**, **h**, *i1* and *i2*, and 10 μm in *i3* and *i4*

There are two major regulatory pathways involved in stem cell determination of carpel development: the *WUS*-*AG* pathway (mainly including *LFY*, *WUS*, and *AG*) and the *KNOX*-*I* pathway (mainly including *STM* and *KNAT2*) [9]. In addition, three *AG*-related MADS-box genes, *SHATTERPROOF1* (*SHP1*), *SHP2*, and *SEEDSTICK* (*STK*), also redundantly control carpel and ovule identities [39, 40]. To gain insight into molecular mechanisms underlying defective carpel development in the *atring1a;atring1b* mutant, we performed quantitative RT-PCR (qRT-PCR) to investigate the expression profiles of these genes in the floral buds of *atring1a;atring1b* compared to WT. *LFY* showed a dramatic increase in expression, whereas the more downstream regulators *WUS* and *AG* were reduced in expression in the mutant (Fig. 5a). While *SHP1* and *SHP2* were unaffected, *STK* also showed reduced expression. In sharp contrast, *KNOX-I* pathway genes, including *STM*, *BP*, *KNAT2*, and *KNAT6*, all showed increases in expression in the mutant (Fig. 5a).

To further dissect spatial and temporal expression patterns of some key regulatory genes, we introgressed the corresponding promoter::*GUS* reporters into the *atring1a;atring1b* mutant. Histological staining revealed that *LFY*::*GUS* is undetectable in WT flower sections (Fig. 5b) but is ectopically expressed in developing carpel and placenta, anthers, and additional organs between petal and carpel in the *atring1a;atring1b* mutant (Fig. 5c). The *WUS*::*GUS* reporter showed specific expression in the floral meristem and developing nucelli similar to WT (Fig. 5d) and *atring1a;atring1b* (Fig. 5e). The *AG*::*GUS* reporter showed weak expression in stamens, and very strong expression in the placenta and ovules inside the gynoecium in WT (Fig. 5f). In the *atring1a;atring1b* mutant, *AG*::*GUS* expression is drastically reduced in placenta and ovules, but still appears in ectopic stigma papillae of carpelloid sepals (Fig. 5g). It is known that LFY and WUS bind independently at the second intron of *AG* and cooperate to activate *AG* expression, but neither LFY nor WUS alone appears to be sufficient to activate *AG* [41]. Consistently, our data showed that a higher level of *LFY* but a lower level of *WUS* failed to elevate *AG* expression in the *atring1a;atring1b* mutant. To provide further insight, we analyzed the expression of *CLV3,* which is known to restrict *WUS* expression to the SAM and FM [42]. The *CLV3*::*GUS* reporter displayed a strict FM expression in WT (Fig. 5h) as expected. In the *atring1a;atring1b* mutant, however, *CLV3*::*GUS* showed a drastic increase of expression in FM as well as ectopic expression in carpel primordia, very young carpels, the placenta region, stigmatic tissue, and the nucellus of ovules (Fig. 5i). The high level of CLV3 likely restricts *WUS* expression in the *atring1a;atring1b* mutant. Our interest further turns to *KNOX-I* genes. Examination of *KNOX-I* genes using *STM::GUS*, *KNAT2::GUS*, and *BP::GUS* reporters revealed that they are ectopically expressed at high levels broadly in young developing gynoecia and in placental tissues, ovules, and carpelloid tissues in the *atring1a;atring1b* mutant as compared to their highly restricted expression in WT (Fig. 5j–o).

Taken together, both our qRT-PCR and reporter gene analyses indicate that the *KNOX-I* genes, but neither *WUS* nor *AG*, are derepressed in *atring1a;atring1b,* and that the spatiotemporal pattern of ectopic *KNOX-I* gene expression correlates with the mutant carpel phenotype.

### Genetic evidence for a pivotal role of AtRING1 in *KNOX-I* suppression during carpel development

**Fig. 6:**
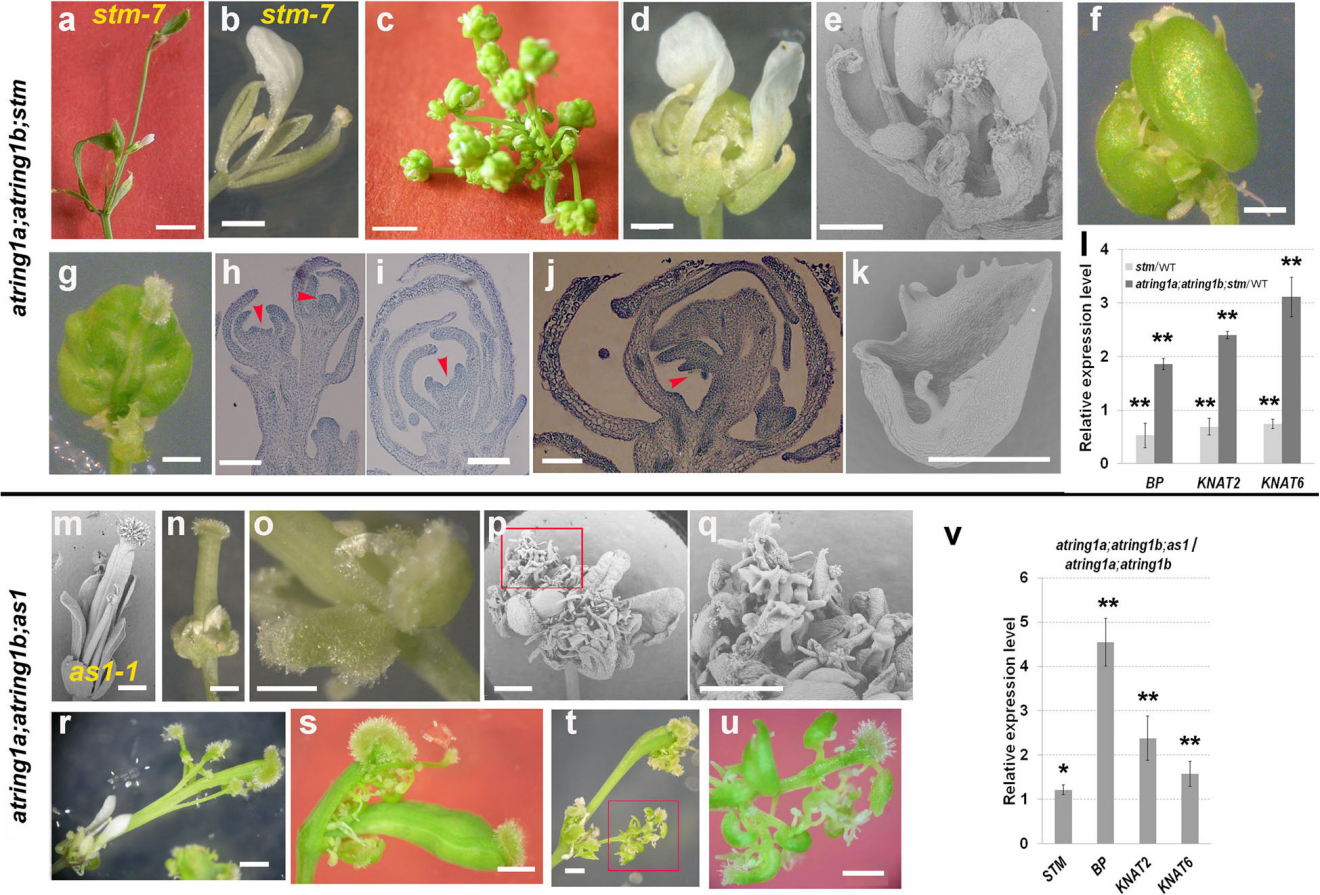
Phenotype analysis of *atring1a;atring1b;stm-7* and *atring1a;atring1b;as1* triple mutant during carpel development. **a**–**l** Phenotype analysis of *atring1a;atring1b;stm-7* triple mutant. **a** Inflorescence stem with clusters of rosette-like leaves in *stm-7* mutant. **b** Absence of carpel within the central whorl of a typical *stm-7* flower. **c** Flower replaces leaf development in the inflorescence of *atring1a;atring1b;stm-7* triple mutant. **d**, **e** A typical triple mutant flower at maturation (**d**) displays defective and unclosed carpel development (**e**). **f** Unclosed carpel. **g** Closed carpel. **h**–**j** Longitudinal section shows defective carpel development (*arrowheads*) in early stages of triple mutant. **k** Carpelloid sepal in triple mutant. **l** Expression levels of *KNOX-I* genes in *atring1a;atring1b;stm-7* triple mutant as compared with *stm-7* mutant (Student’s *t* test, **p < 0.01). Error bars represent SD from three biological replicates. **m**–**v** Flower phenotype analysis of *atring1a;atring1b;as1-1* triple mutant. **m** *as1-1* flower harboring slightly shorter outer floral organs. **n** Outgrowth of outer floral organs is severely repressed. **o** Stigmatic papilla-like structures develop from the sepal margin. **p**, **q** Overproliferated placenta-like outgrowths extend from inside the sepals. **q** Close-up view of **p. r** Several style-stigma structures grow from the side of the central gynoecium. **s** A complete gynoecium grows out from within another unfused one. **t**, **u** Typical *atring1a;atring1b;as1-1* flower producing two floral axes. Reiterations of carpels, ovules, or stigmatic tissues occur along the floral axis (**u**). **v** Elevated *BP* and *KNAT2* expression in the floral buds of *atring1a;atring1b;as1-1* triple mutant compared with *atring1a;atring1b* double mutant detected by qRT-PCR (Student’s *t* test, **p < 0.01). Error bars represent SD from three biological replicates. Bar = 500 μm, except 1 cm in **a** and **c**, 50 μm in **h**–**j**, and 1 mm in **s**–**u**

To directly evaluate the role of *KNOX-I* genes in *atring1a;atring1b* mutant carpel phenotype determinacy, we generated an *atring1a;atring1b;stm-7* triple mutant (Additional file 2: Figure S2). The weak mutant allele *stm-7* contains a transfer DNA (T-DNA) insertion in the second intron of the *STM* locus (Additional file 2: Figure S2) and displays defective inflorescence development, a reduced number of outer floral organs, and no central carpel (~4.2 sepals, ~1 petals, ~1.5 stamen, and 0 carpels, *n* = 17) (Fig. 6a and b, Additional file 2: Figure S2) [43]. Interestingly, we found that *atring1a;atring1b* partially rescues the morphological architecture and floral phenotype of *stm-7*. For instance, the *atring1a;atring1b;stm-7* triple mutant produces inflorescences without obvious whorled phyllotaxy (Fig. 6c) replacing the “aerial rosettes” phenotype with repeated “inflorescence-vegetative”-type development in *stm-7* (Fig. 6a, Additional file 2: Figure S2). The triple mutant flower (~4.2 sepals, ~4.4 petals, ~2.4 stamen, and 3 separate carpels, *n* = 17) with delayed fourth whorl development frequently displays unfused carpels with few defective ovules (Fig. 6d to f, h to j). More rarely, closed carpels can be observed in the central whorl (Fig. 6g). Additionally, homeotic conversions occur frequently, with ~100% of sepals showing carpelloid-like structures (Fig. 6k). It is known that *KNOX-I* genes have redundant functions; for instance, ectopic expression of *KNAT2* and *BP* can suppress *stm* flower phenotypes to various extents [9]. Therefore, we compared by qRT-PCR the expression levels of the other *KNOX-I* genes in floral buds between the *atring1a;atring1b;stm-7* triple mutant and the *stm-7* single mutant. Our data showed that expression levels of *BP*, *KNAT2*, and *KNAT6* are elevated in the triple mutant (Fig. 6l), suggesting that their derepression by *atring1a;atring1b* and their redundant function with *STM* possibly accounts for some phenotypes observed in the *atring1a;atring1b;stm-7* triple mutant. Lastly, we generated the *atring1a;atring1b;bp-1* triple mutant, which displays characteristics of the *atring1a;atring1b* double mutant with the exception of downward-curled pedicel growth similar to *bp-1* (Additional file 3: Figure S3), indicating a specific role of *BP* in determining proper growth of floral pedicels.

It is previously known that the MYB-family transcription factor AS1 represses the expression of *BP*, *KNAT2*, and *KNAT6* in a PRC1/PRC2-associated manner [44, 45]. Investigation of the *atring1a;atring1b;as1-1* triple mutant during the reproductive stage (Additional file 4: Figure S4) revealed that the *as1-1* mutation further enhances the *atring1a;atring1b* phenotype (Fig. 6 m–u). Expansion of outer floral organs (except carpels) was severely inhibited, resulting in protruding gynoecia in *atring1a;atring1b;as1* flowers (Fig. 6n and o), which is in agreement with the higher expression of *BP* and *KNAT2* in the flower buds in *atring1a;atring1b;as1-1* as compared to *atring1a;atring1b* (Fig. 6v). All sepals from the *atring1a;atring1b;as1-1* triple mutant showed carpelloid-like structures (Fig. 6o) with some producing naked placenta-like structures from the inner position (Fig. 6p and q). This latter observation is consistent with strong expression of *AS1* at the inner surface of WT sepal primordia [46]; loss of *AS1* likely enhances expression of *KNOX-I* genes and indeterminate growth in *atring1a;atring1b;as1-1*. Development of whorl 4 in the *atring1a;atring1b;as-1* triple mutant displayed severe pleiotropic phenotypes with indeterminate growth. For instance, several style-stigma structures extend from the flank of the central pistil (Fig. 6r), a secondary gynoecium grows outwards from within another unclosed one (Fig. 6s), and spiral reiterations of carpel-like structures margined by ovules are found along style-like structures topped by stigmatic tissues (Fig. 6t and u). Together, these data indicate that *as1-1* enhances flower and carpel phenotypes of the *atring1a;atring1b* mutant through further synergistic increases in *KNOX-I* gene expression. But an alternative explanation with a small possibility would be that AS1 might act on other unknown carpel-controlling genes which are competitively regulated by *KNOX-I* genes.

### Carpel development in the *atring1a;atring1b* mutant still requires *WUS* function

**Fig. 7:**
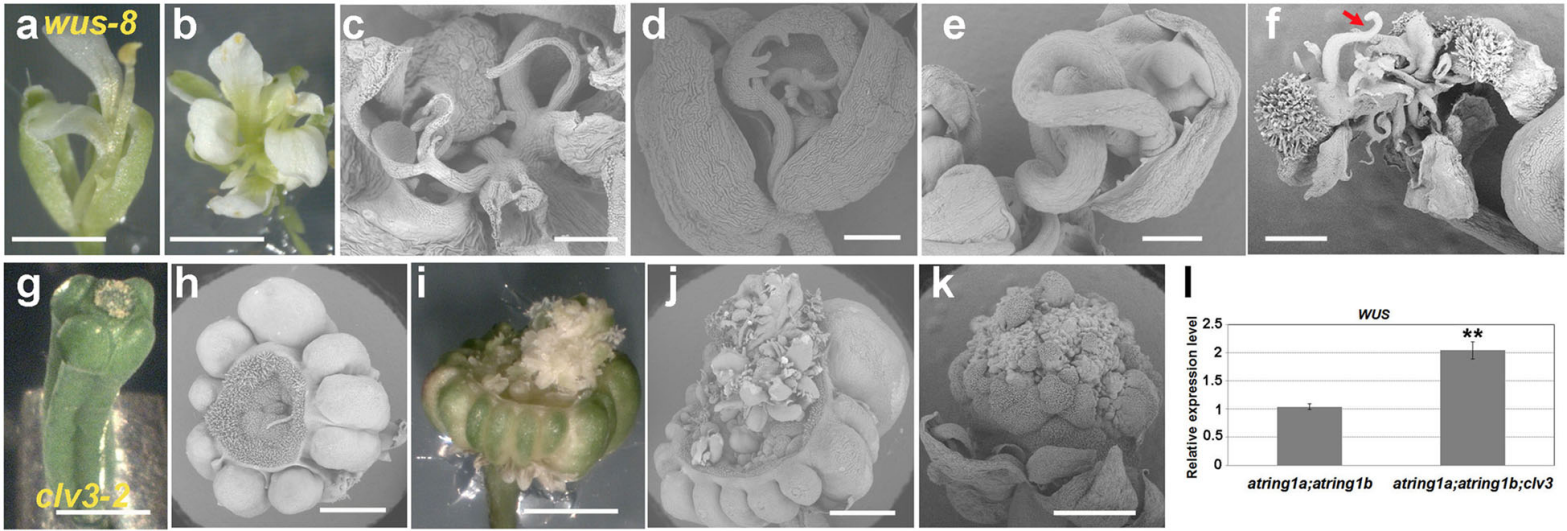
Phenotype analysis of *atring1a;atring1b;wus-8* and *atring1a;atring1b;clv3-2* triple mutants. **a**–**f** Phenotype of *atring1a;atring1b;wus-8* triple mutant flower. **a** A typical *wus* flower showing reduced floral organs and absence of carpel. **b** A typical *atring1a;atring1b;wus-8* triple mutant flower showing increased number of sepals and petals, but still lacking central carpel. **c** Absence of central carpel but production of filamentous extra organs in *atring1a;atring1b;wus-8* triple mutant flower. **d** Filamentous organ produced from the base of sepal. **e** Filamentous organ with a long branch curled inside a carpelloid sepal. **f** Ectopic carpel-like structure developed from outer whorls of *atring1a;atring1b;wus-8* triple mutant flower. *Arrow* indicates a branch mimicking an ovule outgrowth. **g**–**k** Gynoecium phenotype of *atring1a;atring1b;clv3-2* triple mutant flower. **g** A typical *clv3-2* gynoecium fused with four carpels. **h** Increased carpel number and (**i**) overproliferated stigmatic papilla-like structures in *atring1a;atring1b;clv3-2* triple mutant. **j** Abundant carpel-like structures outgrown from inside of gynoecium. **k** Stigmatic papilla-like structures overproliferating at top of gynoecium. Bars = 1 mm, except 200 μm in **c**–**f. l** Elevated *WUS* expression in floral buds of *atring1a;atring1b;clv3-2* triple mutant compared with segregated *atring1a;atring1b* sibling detected by qRT-PCR (Student’s *t* test, **p < 0.01). Error bars represent SD from three biological replicates

It is well known that *STM* and *WUS* act independently but complementarily in the maintenance of proper shoot apical meristem activity [8]. Although neither *WUS* nor *AG* is repressed by AtRING1a/AtRING1b, it remains unclear whether the *WUS*-*AG* pathway has a role in determinacy of the *atring1a;atring1b* mutant phenotype. To investigate the role of *WUS* in the *atring1a;atring1b* mutant, we constructed an *atring1a;atring1b;wus-8* triple mutant (Additional file 5: Figure S5). The *wus-8* mutant displays a typical loss-of-function *wus* phenotype, as previously described [47], e.g., premature termination of shoot and floral meristem activities, absence of carpels, and reduced numbers of other floral organs (~2.6 sepals, ~2 petals, ~0.1 stamen, and 0 carpels, *n* = 10) (Fig. 7a). The *atring1a;atring1b;wus-8* triple mutant shows higher numbers of outer floral organs and the absence of central carpels (~5.3 sepals, ~13.2 petals, ~3.8 stamens/petaloid stamens, and 0 carpels, *n* = 10) (Fig. 7b and c), indicating that loss of *AtRING1* fails to rescue *wus-8* in carpel development. In this triple mutant flower, additional filamentous organs were observed at whorls interior to the sepals (Fig. 7c–e). Some of these organs can develop into carpel-like structures with branching filamentous structures that mimic ovules but lack nucellus and integument differentiation (Fig. 7f). These ascribed phenotypes of the *atring1a;atring1b;wus-8* mutant flowers are closely similar to those previously reported for the flowers of the *STM* ^*OE*^;*wus* plants that overexpress *STM* in the *wus* mutant [9].

To evaluate an effect of *WUS* overexpression in the *atring1a;atring1b* mutant, we constructed the *atring1a;atring1b;clv3-2* triple mutant (Additional file 6: Figure S6). CLV3 polypeptide acts as a small secreted ligand, binding to CLV1 and CLV2-CORYNE (CRN) heteromeric receptors to restrict the domain of *WUS* expression (reviewed in [48]). *clv* mutants with increased *WUS* expression accumulate excess numbers of undifferentiated cells in both shoot and floral apices, leading to shoot fasciation, enlarged floral meristems, and supernumerous carpels (Fig. 7g, Additional file 6: Figure S6, [49]). Remarkably, as shown in Fig. 7h–j, the *atring1a;atring1b;clv3-2* triple mutant displays sharply increased carpel numbers (~7.5, *n* = 37) as compared to either *atring1a;atring1b* (~3.2, *n* = 14) or *clv3-2* (~4.4, *n* = 34). In addition, papillae overproliferation on stigma and inside gynoecia is also more severe in the triple mutant (Fig. 7i–k). Thus, loss of *CLV3* releases *WUS* suppression, further enhancing carpel indeterminacy of the *atring1a;atring1b* mutant. Indeed, qRT-PCR analysis confirmed that *WUS* expression is higher in *atring1a;atring1b;clv3-2* than in *atring1a;atring1b* (Fig. 7l).

Taken together, our data indicate that while derepression of *KNOX-I* genes in *atring1a;atring1b* induces carpel indeterminacy, central carpel development still depends on *WUS*, and increased *WUS* can further enhance carpel indeterminacy in the mutant. Thus, *KNOX-I* and *WUS* genes likely act independently but complementarily in normal carpel development in a similar way in which *STM* and *WUS* function in maintenance of shoot development [8].

## Discussion

### PRC1 RING finger genes are broadly expressed, with *AtRING1b* and *AtBMI1c* specifically showing parental imprinting

Loss of either AtRING1a or AtRING1b does not impact plant development, but the *atring1a;atring1b* double mutant displays pleiotropic phenotypes throughout vegetative and reproductive stages, indicating their redundant functions in plant development. Detailed expression analysis using *GUS* reporter constructs confirmed very similar and wide-ranging expression patterns of both *AtRING1a* and *AtRING1b* during the plant life cycle. In the early vegetative stage, embryo-like structures develop in the SAM and young leaves, and *pkl*-like taproot and twist rosette leaves are also found in *atring1a;atring1b,* which is in agreement with strong expression of both *AtRING1a* and *AtRING1b* found in SAM, RAM, young leaves, and vasculatures. Proceeding to the reproductive phase, *AtRING1a* and *AtRING1b* were first detected in the inflorescence apex and various floral organ primordia, which is consistent with the high number of floral organs and homeotic conversions observed in *atring1a;atring1b. AtRING1a/AtRING1b* expression gradually decreases or completely ceases as surrounding floral organs expand, suggesting the highest activity for *AtRING1a/AtRING1b* in proliferating/rapidly dividing tissues. Lastly, both *AtRING1a* and *AtRING1b* are expressed during various stages of carpel, ovule, and embryo development, which is consistent with the homeotic conversions of replum/ovule-to-carpel and defective ovule formation observed in *atring1a;atring1b* mutant gynoecia.

The major difference between *AtRING1a* and *AtRING1b* is the specific expression of *AtRING1b*, but not *AtRING1a*, in endosperm. So far, most known examples of imprinted genes are confined to the endosperm in higher plants. For instance, the PRC2 component genes *FIS2* and *MEA* display maternally biased expression in the endosperm [31, 50, 51]. Moreover, all known plant genes with imprinted expression depend on differential DNA methylation, PRC2 activity, or both. Investigation based on reciprocal crosses of either the *AtRING1b::AtRING1b-GUS* reporter or the *AtBMI1c*::*AtBMI1c*-*GUS* reporter with WT, *met1-3*, or *fis2* showed that they display preferentially maternal expression in the endosperm, and that both *AtRING1b* and *AtBMI1c* are regulated by CG DNA methylation independent of the FIS2-PRC2 complex. Loss of function of each FIS2-PRC2 component (i.e., *MEA*, *FIE*, *FIS2*, and *MSI1*) causes endosperm overproliferation without fertilization, embryo abortion, and seed lethality [21]. Neither *atbmi1c* nor *atring1b* single mutants show a visible phenotype during endosperm development, indicating that both have redundant functional homologs or that PRC1 might have only a minor effect and separate function from FIS2-PRC2 during endosperm development.

### Ovule development is impaired in the *atring1a;atring1b* mutant

Both *AtRING1a* and *AtRING1b* display strong expression throughout ovule and ES development, indicating their potential importance in megasporogenesis and megagametogenesis. Indeed, the *atring1a;atring1b* mutant displays broad abnormalities during ovule development, ranging from ovule morphology and structure to ES formation. On one hand, *AtRING1a* and *AtRING1b* inhibit nucellus overproliferation, but indirectly promote outer integument growth. On the other hand, *AtRING1a/b* regulate ES development by ensuring degeneration of destined megaspores after meiosis. Thus, *AtRING1a/b* coordinate ovule development in both sporophytic and gametophytic phases. Many gametophytic ovule mutants have normal sporophytic tissue structures, but sporophytic ovule mutants usually have abnormal gametophyte development, suggesting that integument and ES development are interdependent processes and that accurate architecture of sporophytic tissue is necessary for successful development of a fully functional gametophyte [52]. For example, *bell1* (*bel1*) ovules develop a single integument-like structure (ILS) taking the place of the two integuments, and fail to produce a normal ES [36, 53, 54]. In *short integuments1* (*sin1*) ovules, both integuments are too short to enclose the nucellus, and the ES does not develop [36, 55]. Therefore, continuous signaling from sporophytic tissue appears necessary to precisely direct gametophyte development. Here, in the *atring1a;atring1b* double mutant, defective outer integument and nucellus growth may cause the arrest of ES development. Alternatively, *AtRING1a/b* might control sporophytic tissue and ES development in a parallel manner. Further investigation through complementation experiments by introducing recombinant genes expressing *AtRING1* under the control of integument-, nucellus-, or ES-specific promoters into the *atring1a;atring1b* mutant may be helpful to address these questions.

### *AtRING1a* and *AtRING1b* control carpel development mainly through repression of *KNOX-I* genes

**Fig. 8:**
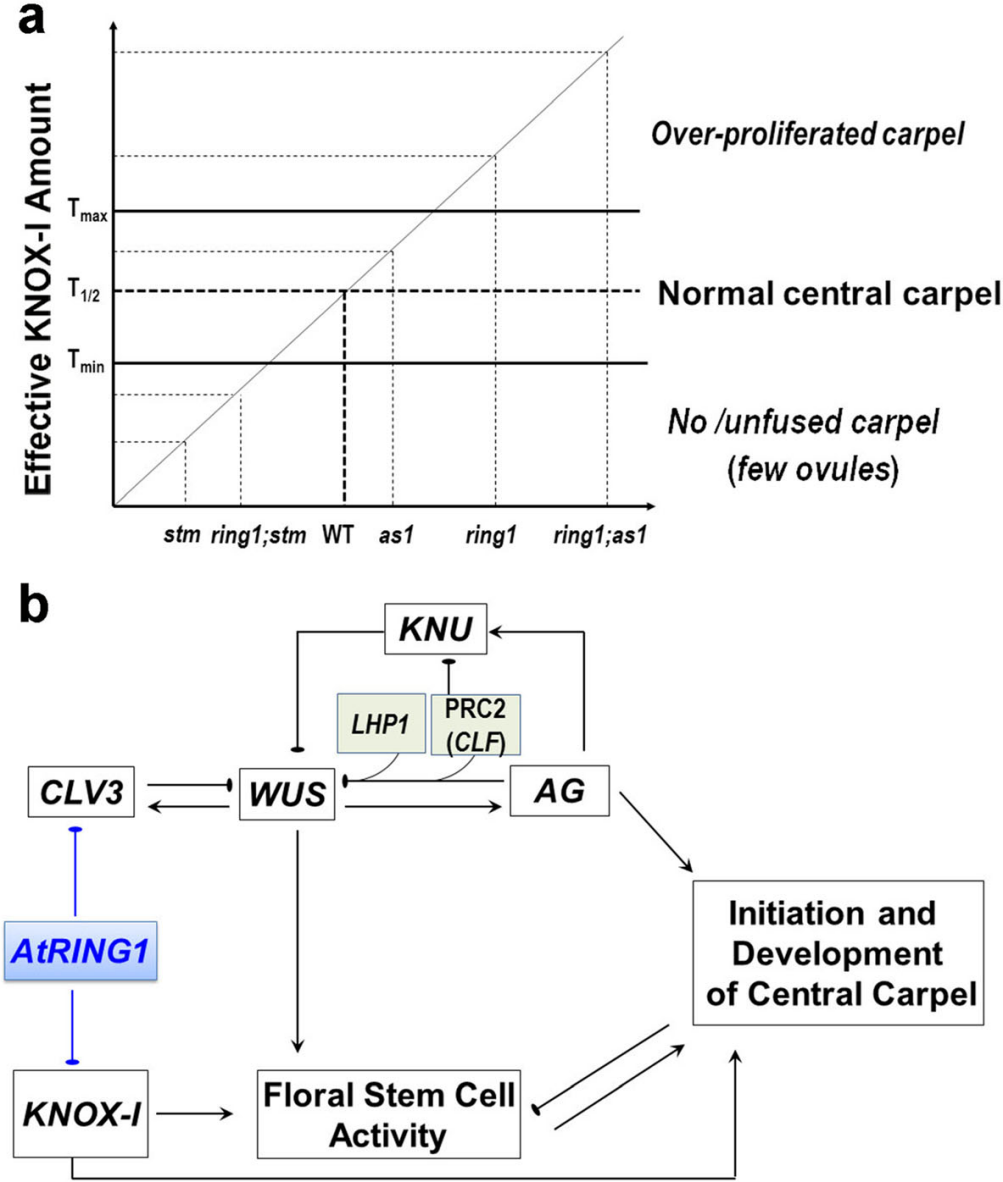
Hypothetical model of AtRING1-mediated *KNOX-I* repression in carpel development. **a** Hypothetical dosage effect of KNOX-I explaining the varied severity of carpel developmental defects observed in the studied mutants. Because different *KNOX-I* genes regulate carpel development with an efficiency of *STM* > *KNAT2* > *BP*, we propose an effective KNOX-I amount (*Y*-axis) by considering KNAT2 = 1/N STM and BP = 1/(N + X) STM, with N > 1, X > 0. T_1/2_ represents the amount for WT carpel development, and T_min_ and T_max_ indicate the minimum and maximum threshold, respectively, for allowing normal carpel development. Mutants with estimated range of effective KNOX-I levels and respective carpel phenotypes are indicated. **b** Hypothetical model of *AtRING1* function within a gene network controlling floral stem cell activity and carpel development. *AtRING1* as well as PRC2 (*CLF*) and *LHP1* are *colored. KNOX*-*I* genes (including *STM*, *KNAT2*, and *BP*), *CLV3*, and the *WUS*-*AG-KNU* feedback loop are indicated. *Arrows* indicate promotion, and *T-shaped bars* indicate repression

In Arabidopsis there are at least two independent and complementary pathways, the *WUS*-*AG* pathway and the *KNOX*-*I* pathway, controlling stem cell activity and carpel development. Several lines of evidence indicate that *AtRING1a* and *AtRING1b* act mainly via repression of the *KNOX*-*I* but not the *WUS-AG* pathway. Firstly, qRT-PCR showed that the expression of *KNOX-I* genes is significantly increased, which is in contrast to the decreased expression of *WUS* and *AG*, in the *atring1a;atring1b* mutant. Notably *LFY,* an upstream regulator of the *WUS*-*AG* pathway, is drastically upregulated in floral buds of *atring1a;atring1b*, which might be associated with floral reversion such as the ”flower-in-flower” phenotype observed in the mutant [28]. Secondly, *GUS* reporter analysis revealed that ectopic *KNOX-I* gene expression occurs in developing carpels, ovules, and carpel-like structures of the *atring1a;atring1b* mutant, whereas *AG* lines display weak GUS staining. Expression of *WUS* in secondary ovules and of *AG* in ectopic papillae of carpelloid-like sepals was observed in the *atring1a;atring1b* mutant, which likely reflects the requirement for *WUS*-*AG* in specifying ovule-carpel identity. Thirdly, ovule-to-carpel conversion observed in the *atring1a;atring1b* mutant is reminiscent of transgenic plants overexpressing *STM* or *KNAT2* described in a previous study [9]. Finally, genetic analysis demonstrates that misexpression of *KNOX-I* genes is important for the defective carpel developmental phenotype observed in *atring1a;atring1b*. Loss of AtRING1 activities partially rescue *stm* architecture and flower phenotype due to release of the other *KNOX-I* genes in the *atring1a;atring1b;stm-7* triple mutant; this resembles *as1* partial rescue of the *stm* phenotype in the *as1;stm* double mutant via upregulation of other *KNOX-I* genes [46]. Furthermore, we found that *as1* can enhance *atring1a;atring1b* flower and carpel phenotypes due to a synergistic derepression of *KNOX-I* genes in the *atring1a;atring1b;as1-1* triple mutant. Thus, repression of *KNOX-I* genes constitutes an important regulatory mechanism in carpel and ovule development, and a dosage-dependent effect of *KNOX-I* genes likely explains the degree of severity on central carpel development defects observed across the mutants studied (Fig. 8a).

Our conclusion that the PRC1 core components AtRING1a and AtRING1b repress the *KNOX-I* but not the *WUS*-*AG* pathway is also in agreement with a previous study carried out using *atring1a;atring1b* mutant seedlings [28]. PRC1 RING finger proteins AtBMI1a and AtBMI1b are also not required for *AG* repression in seedlings [27]. In contrast, it is well known that *AG* is derepressed in *lhp1* and PRC2-related mutant plants, indicating that LHP1 and PRC2 are involved in the developmental switch from SAM to FM. During the reproductive stage, PRC2-mediated H3K27me3 has been shown to play a critical role in repressing *WUS* directly via AG recruitment, or indirectly via competitive displacement by AG at the promoter of the *WUS* repressor *KNU*; together these mechanisms account for floral stem cell termination and carpel development initiation ([19, 20]; Fig. 8b). Our finding that *AtRING1* suppresses *KNOX-I* and *CLV3* provides additional novel information to the gene networks controlling floral stem cell activity and carpel development (Fig. 8b). Stem cells are consumed upon carpel development, yet they are required at the initiation of carpel development; thus, disruption of the stem cell master regulator *WUS* or *STM* leads to the absence of central carpel development. Our analyses of *atring1a;atring1b* together with the *atring1a;atring1b;stm-7* and *atring1a;atring1b;as1-1* triple mutants clearly establish a key role of *KNOX-I* suppression in maintenance of carpel and ovule determinacy. Interestingly, derepression of *KNOX-I* alone is insufficient, and proper carpel initiation depends on the *WUS-AG* pathway as evidenced by the absence of carpel development in the *atring1a;atring1b;wus-8* triple mutant and the polycarpous proliferation in the *atring1a;atring1b;clv3-2* triple mutant. Although direct genetic interaction between *AtRING1* and *AG* is unexamined so far, it is well established that *CLV3* and *WUS* regulate carpel development through *AG*, which is the key regulator in determination of carpel identity [13, 14]. Thus, it is reasonable to conclude that the *WUS*-*AG* and *KNOX-I* pathways act independently and complementarily in regulation of carpel development. Similarly, it is well known that *WUS* and *STM* act independently and complementarily in maintaining vegetative shoot development [56]. These independent and complementary pathways, together with the finding that AtRING1 and PRC2 regulate various pathway genes with different specificity, may mean that it is advantageous for stem cells to integrate diverse developmental and environmental cues to cope with plant developmental plasticity. It is also reasonable to speculate that *AtRING1*-mediated suppression of *CLV3* may provide a link coordinating the *WUS*-*AG* pathway with the *KNOX-I* pathway in the regulation of floral stem cell activity and carpel development (Fig. 8b). Meanwhile, PRC2-mediated H3K27me3 involvement in suppression of *KNOX-I* genes during vegetative growth is well described [57]. Future studies are necessary to determine to which extent PRC2 repression is involved in floral stem cell termination and carpel development and whether it is associated with AtRING1 function.

## Conclusion

Our study provides important information about tissue-specific expression patterns and unravels a key role of the PRC1 core component genes *AtRING1a* and *AtRING1b* in suppression of *KNOX*-*I* genes, which independently but complementarily act together with the *WUS-AG* pathway to determine floral stem cell proliferation and termination during flower carpel development.

## Methods

### Plant materials

Mutant alleles *fis2* (SALK_009910), *met1-3* (CS16394), *stm-7* (N409575, GK-100F11), *wus-8* (SAIL_150_G06), *as1-1* (CS146), *clv3-2* (CS8066), and *bp-1* (CS30) and GUS reporters *LFY*::*GUS* (N9776) and *CLV3*::*GUS* (N9610) were obtained from the Arabidopsis Biological Resource Center (ABRC, Columbus, OH, USA) or the Nottingham Arabidopsis Stock Centre (NASC, Loughborough, UK). The *atring1a;atring1b* double mutant and *atring1a;atring1b;as1-1* triple mutant have been described previously [23, 28]. *WUS*::*GUS* and *KNOX*::*GUS* (*STM*::*GUS*, *BP*::*GUS*, and *KNAT2*::*GUS*) reporters and the *atring1a;atring1b* double mutant background have also been described previously [28]. Seeds were surface sterilized (70 and 95% ethanol for 10 min, respectively) and plated on Murashige and Skoog (MS) medium (MS salts, 0.9% sucrose, pH 5.7, 0.9% bactoagar). After stratification at 4 °C for 2 days in the dark, plates were incubated in a growth chamber at 22 °C under a 16-h light/8-h dark regime. After 10 days, the seedlings were transferred to soil and grown under the same conditions.

### Generation of mutant combinations

To generate the *atring1a;atring1b;stm* triple mutant, a *stm-7* ^-/+^ heterozygote was crossed to an *atring1a* ^-/+^;*atring1b* ^-/-^ plant. The *atring1a* ^-/+^ *;atring1b* ^-/-^ *;stm* ^-/+^ triple mutant was obtained in the F2 generation by genotyping (the genotyping primers are listed in Additional file 7: Table S1), which produced four segregating phenotypes: WT, *stm*, *atring1a;atring1b*, and the triple mutant phenotype in the F3 generation. Plants were analyzed by PCR to select the *atring1a;atring1b;stm-7* homozygous mutant, which exhibits an *atring1a;atring1b*-like phenotype in the vegetative stage and partially rescued *stm-7* flower phenotype in the reproductive stage.

To generate the *atring1a;atring1b;wus* triple mutant, a *wus-8* ^-/+^ heterozygote was crossed to an *atring1a* ^-/+^;*atring1b* ^-/-^ plant. The *atring1a* ^-/+^ *;atring1b* ^-/-^ *;wus* ^-/+^ triple mutant was obtained in the F2 generation by genotyping (the primers are listed in Additional file 7: Table S1), which produced four segregated phenotypes: WT, *wus*, *atring1a* ^-/+^;*atring1b* ^-/-^, and the triple mutant phenotype in the F3 generation. Plants were analyzed by PCR to select the *atring1a;atring1b;wus-8* homozygous mutant, which exhibits an *atring1a;atring1b*-like phenotype in the vegetative stage and no central carpel development in the reproductive stage.

To generate the *atring1a;atring1b;clv3-2* triple mutant, a *clv3-2* homozygote was crossed to an *atring1a* ^-/+^ *;atring1b* ^-/-^ plant. The *atring1a* ^-/+^ *;atring1* ^-/-^ *;clv3-2* ^-/-^ triple mutant displaying the typical *clv3* phenotype was obtained in the F2 generation by genotyping, and it produced three segregating phenotype populations: *clv3*, *atring1a;atring1b*, and the triple phenotype in the F3 generation. Plants were analyzed by PCR to select the *atring1a;atring1b;clv3-2* homozygous mutant, which exhibits an *atring1a;atring1b*-like phenotype in the vegetative stage and proliferated stigma in the reproductive stage.

Generation of *atring1a;atring1b;bp-1* was similar to that of *atring1a;atring1b;clv3-2*. Firstly, an *atring1a* ^-/+^ *;atring1* ^-/-^ *;bp* ^-/-^ triple mutant displaying the typical *bp* mutant phenotype was obtained in the F2 generation. Three phenotypes including *bp*, *atring1a;atring1b*, and the triple mutant phenotype were segregated in the F3 generation. Genotyping PCR was performed to identify the homozygous triple mutant *atring1a;atring1b;bp-1*, which displays an *atring1a;atring1b*-like phenotype with a downward pedicel.

### Plasmid construction

For *AtRING1b*::*AtRING1b*-*GUS* construction, the DNA fragment containing the upstream promoter and full genomic DNA region of the *AtRING1b* gene lacking the stop codon (–1586 to +2657 bp) was amplified from Arabidopsis genomic DNA using specific primers, digested using *Sal*I and *Bam*HI, and cloned into pBI101 to create a GUS reporter gene fusion. For *AtBMI1c*::*AtBMI1c*-*GUS* construction, the AtBMI1c genomic region without the stop codon plus its upstream promoter (–2126 to +2246 bp) was obtained using specific primers, digested, and cloned into pBI101, similar to *AtRING1b*::*AtRING1b*-*GUS* construction. These binary vectors were introduced into *Agrobacterium tumefaciens* GV3101 and transformed into Arabidopsis plants by the floral dip method [58]. Primers for plasmid construction are listed in Additional file 7: Table S1.

### RNA isolation and qRT-PCR

Total RNA was isolated using the NucleoSpin RNA Plant kit (Macherey-Nagel, Düren, Germany). qRT-PCR was performed on a LightCycler 480II (Roche), as recommended by the manufacturer. Reaction volumes were scaled to 10 μl final volume and comprised 5 μl SYBR Green PCR master mix (Roche), 2 μl primer mix, and 1 μl template cDNA. All reactions were repeated in triplicate. Protein phosphatase 2 (PP2A) was used as an internal control. The primers for qRT-PCR are listed in Additional file 7: Table S1. Three biological replicates were performed; the original data together with the statistical analysis are given in Additional file 8: Table S2.

### Histochemical staining and imprinting analysis

For GUS (β-glucuronidase) staining, seedlings or floral buds were submerged in 90% acetone for 30 min on ice, washed twice with 1 M sodium phosphate buffer (pH 7.2) for 15 min at room temperature, and subsequently incubated in X-Gluc solution at 37 °C for 1–12 h depending on the desired staining intensity. Thereafter, seedlings were incubated overnight in 70% ethanol at 4 °C. The standard X-Gluc solution contains 0.1 M sodium phosphate buffer (pH 7.2), 0.5 mM Fe(CN)_2_, 0.5 mM Fe(CN)_3_, 0.1% Tween-20, and 2 mM 5-bromo-4-chloro-3-indolyl-β-d-glucuronide (X-Gluc). For imprinting analysis, GUS staining was performed using siliques collected at 3 days after pollination. All crosses in this study were performed at identical growth conditions and timings. Crossing success was confirmed in the F1 progeny by genotyping using GUS-specific primers and the respective mutant primes (Additional file 7: Table S1).

### Microscopy

SEM images were taken using a Hitachi S-3400 N microscope (Hitachi High-Technologies Europe, Krefeld, Germany). Bright-field photographs of individual flowers were taken using a dissecting microscope (Leica, Germany). For DIC observation, dissected pistils were cleared and mounted in chloral hydrate:glycerol:H_2_O (8:2:1, w:v:v) overnight and observed using a DIC microscope (Zeiss, Germany). CLSM observation of ovules was performed according to the method previously described [7] with slight modifications. Dissected pistils with exposed ovules were immersed in fixative (4% glutaraldehyde, 0.1 M phosphate-buffered saline (PBS), pH 7.0), vacuum infiltrated for 30 min, and then fixed overnight at room temperature. Following fixation, the tissue was dehydrated through a graded ethanol series with about 10–20 min per step. After dehydration, the tissue was cleared in benzyl benzoate:benzyl alcohol (2:1, v/v) for 1 or 2 days. Individual ovules were dissected, mounted with immersion oil (high viscosity), and observed using a Zeiss LSM 700 META laser scanning microscope (Zeiss, Germany) with a 488-nm argon laser and an LP 530 filter.

### Paraffin section and in situ hybridization

For paraffin sectioning, samples were fixed in formaldehyde:glacial acetic acid: 70% ethanol (1:1:18, v:v:v) and dehydrated in a graded butanol/ethanol series. Tissues were embedded in paraffin (Leica, Germany) and microtome sections (10 μm) applied onto silane-coated slides. Sections were deparaffinized in xylene and dehydrated through a graded ethanol series before toluidine blue staining. Sections were observed under a Nikon Eclipse 800 microscope. In situ hybridization was performed according to standard protocols [59]. For the preparation of the *AtRING1a* probe, a fragment containing 486 bp of the 5′ region of *AtRING1a* was amplified using specific primers (Additional file 7: Table S1) and cloned into the pGEM-T Easy vector (Promega, Madison, WI, USA).

## Additional files

Additional file 1: Figure S1. Expression pattern of *AtRING1a* in the reproductive stage examined by in situ hybridization using a specific *AtRING1a* probe. (A) Leaf primordia. (B) and (C) SAM. (D) FM. (E) Young flower. (F) Developing ovule. (G) Developing ES. (H) Developing seed in globular stage. (I) Heart stage. (J) Mature green stage. Bars = 50 μm in (A)–(J). (JPG 951 kb)
Additional file 2: Figure S2. Plant architecture of *atring1a;atring1b;stm-7* mutant. (A) Adult plant of *stm-7* mutant. (B) Adult plant of *atring1a;atring1b;stm-7* mutant. Bars = 2 cm. (C) Schematic structure of the *stm-7* mutant allele GK-100 F11 containing a transfer DNA (*T-DNA*) insertion in the second intron of *STM. Gray box* represents UTR, *black box* represents exon, and *line* represents intron. (JPG 623 kb)
Additional file 3: Figure S3. Phenotype analysis of *atring1a;atring1b;bp-1* plant. (A) Adult *bp-1* plant (Ler). (B) Adult *bp-1* control. (C) Adult *atring1a;atring1b;bp-1* plant. The control *bp-1* ^*-/-*^ mutant (B) and *atring1a;atring1b;bp-1* triple mutant (C) are derived from the same F2 generation of *atring1a* ^*-/+*^ *;atring1b* ^*-/+*^ *;bp-1* ^*-/-*^. *Insets* indicate the close-up view of downward branch related to corresponding mutant. Bars = 2 cm in (A)–(C) and 1 mm in the corresponding insets. (JPG 711 kb)
Additional file 4: Figure S4. Phenotype analysis of *atring1a;atring1b;as1-1* mutant. (A) Adult plant architecture of *as1-1* mutant. (B) Adult plant architecture of *atring1a;atring1b;as1-1* mutant. Bars = 2 cm. (JPG 665 kb)
Additional file 5: Figure S5. Phenotype analysis of *atring1a;atring1b;wus-8* triple mutant. (A) Adult plant of *wus-8* mutant. (B) Adult plant of *atring1a;atring1b;wus-8* triple mutant. (C) Close-up view of (B), showing the main florescence of *atring1a;atring1b;wus-8* triple mutant. Bars = 2 cm in (A) and (B) and 500 μm in (C). (D) The *wus-8* allele (SAIL_150_G06) harboring a T-DNA insertion in the second intron of *WUS. Gray box* represents UTR, *black box* represents exon, and *line* represents intron. (JPG 599 kb)
Additional file 6: Figure S6. Phenotype analysis of *atring1a;atring1b;clv3-2* triple mutant. (A) Whole plant architecture of *clv3-2* mutant. (B) Whole plant architecture of *atring1a;atring1b;clv3-2* triple mutant. The control *clv3-2* ^*-/-*^ mutant and *atring1a;atring1b;clv3-2* triple mutant are derived from the same F2 generation of *atring1a* ^*-/+*^ *;atring1b* ^*-/+*^ *;clv3-2* ^*-/-*^. Bars = 2 cm. (JPG 521 kb)
Additional file 7: Table S1. Primers used for genotyping, plasmid construction, and qRT-PCR. (DOC 86 kb)
Additional file 8: Table S2. Original data and statistical analysis for qRT-PCR analyses. (XLSX 12 kb)

## Abbreviations

AG: AGAMOUS
BP: BREVIPEDICELLUS
CLSM: confocal laser scanning microscopy
CLV: CLAVATA
DIC: differential interference contrast
ES: embryo sac
FIS2: FERTILIZATION INDEPENDENT SEED 2
FM: floral meristem
H3K27me3: histone H3 lysine 27 trimethylation
IM: inflorescence meristem
KNU: KUNCKLES
LFY: LEAFY
LHP1: LIKE HETEROCHROMOTIN PROTEIN1
MET1: DNA METHYLTRANSFERASE1
MMC: megaspore mother cell
PcG: Polycomb group
PRC: Polycomb repressive complex
RAM: root apical meristem
SAM: shoot apical meristem
SEM: scanning electron microscopy
SHP1: SHATTERPROOF1
STK: SEEDSTICK
STM: SHOOT MERISTEMLESS
WUS: WUSCHEL
