# Supplementary figures and images for: Arabidopsis PRC1 core component AtRING1 regulates stem cell-determining carpel development mainly through repression of class I KNOX genes
Source: BMC Biol. 2016 Dec 22;14:112. doi: 10.1186/s12915-016-0336-4 (PMC5178098; doi:10.1186/s12915-016-0336-4)

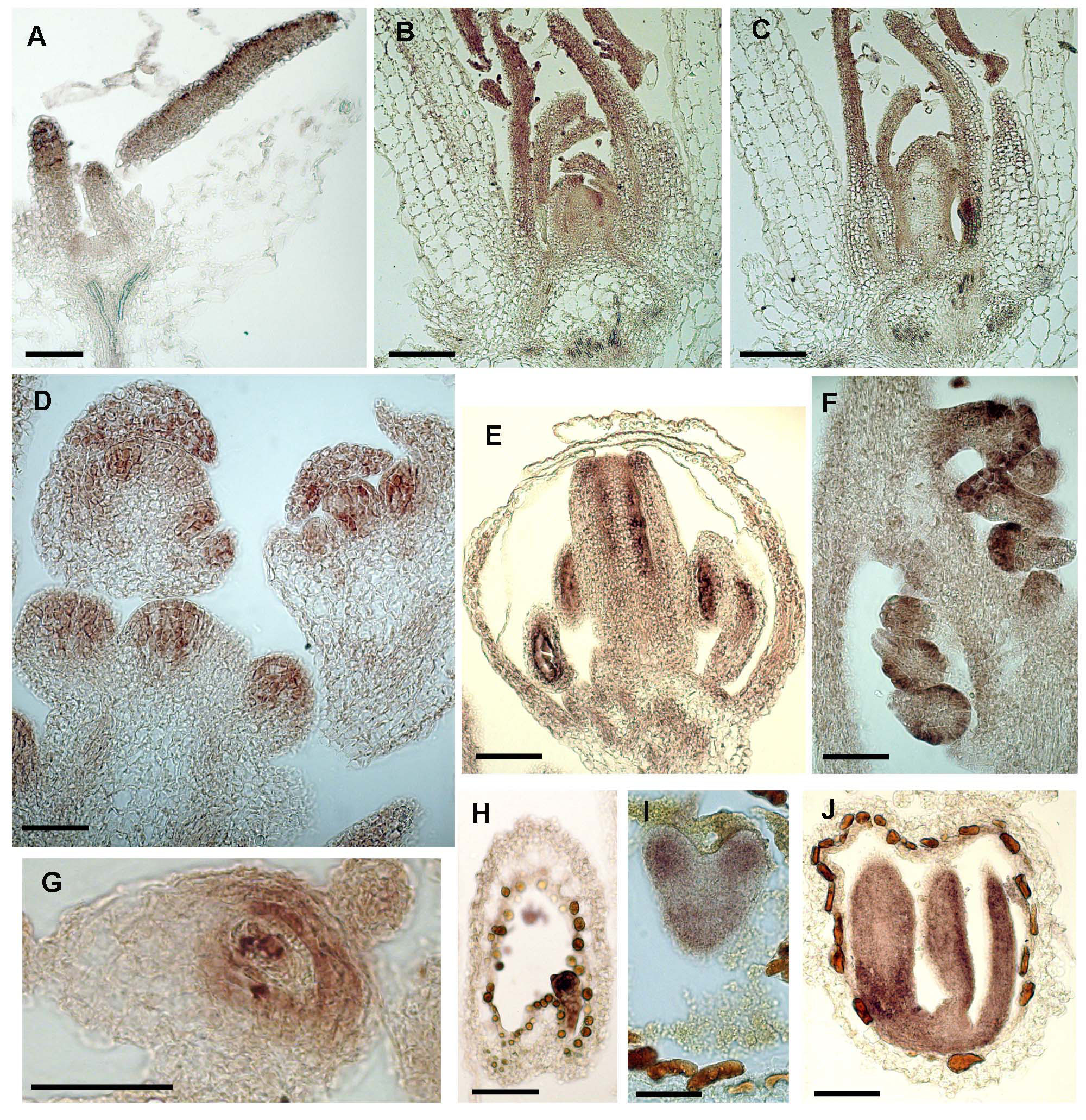

Supplement: Additional file 1: Figure S1. — Expression pattern of AtRING1a in the reproductive stage examined by in situ hybridization using a specific AtRING1a probe. (A) Leaf primordia. (B) and (C) SAM. (D) FM. (E) Young flower. (F) Developing ovule. (G) Developing ES. (H) Developing seed in globular stage. (I) Heart stage. (J) Mature green stage. Bars = 50 μm in (A)–(J). (JPG 951 kb) [file 12915_2016_336_MOESM1_ESM.jpg]

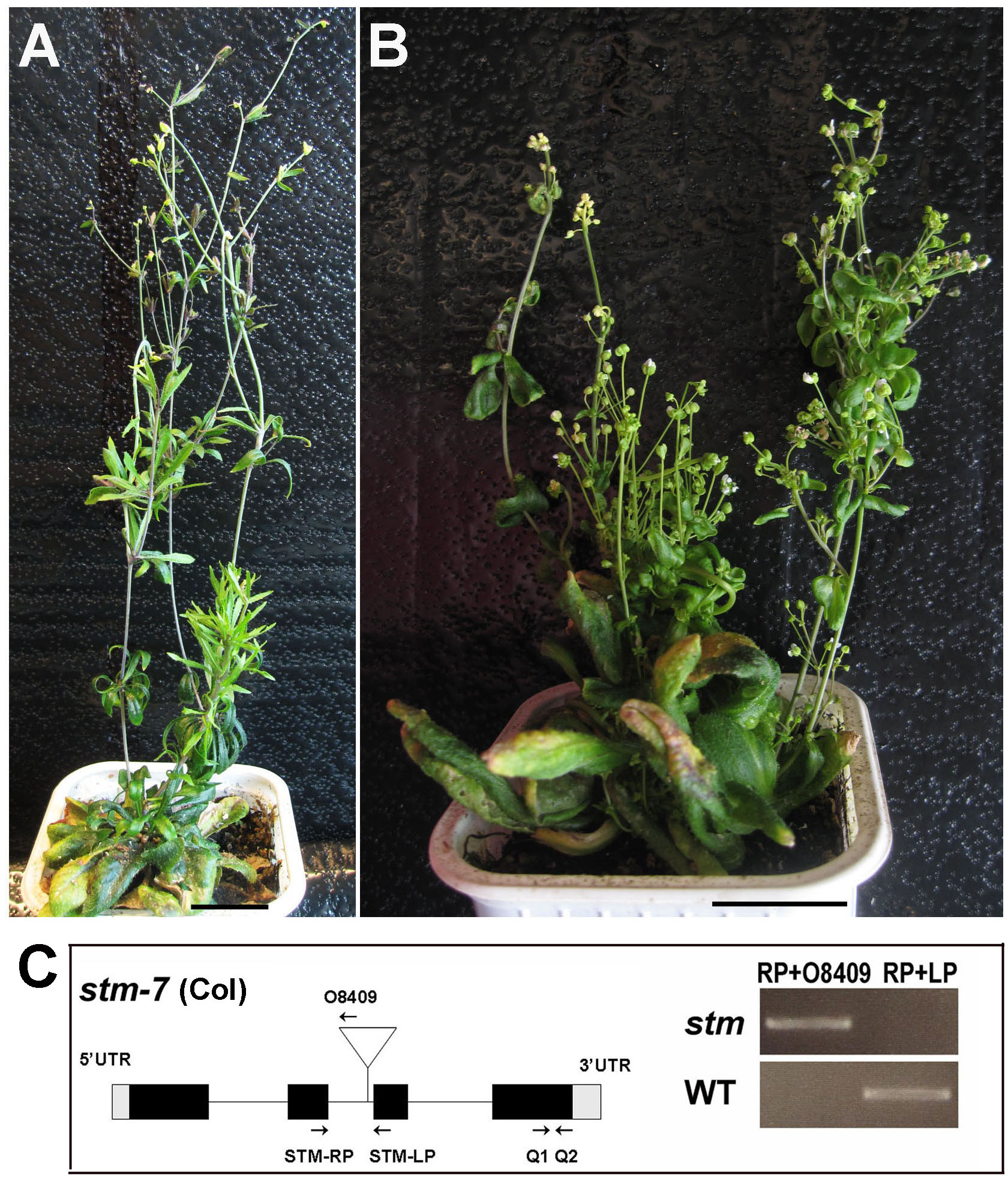

Supplement: Additional file 2: Figure S2. — Plant architecture of atring1a;atring1b;stm-7 mutant. (A) Adult plant of stm-7 mutant. (B) Adult plant of atring1a;atring1b;stm-7 mutant. Bars = 2 cm. (C) Schematic structure of the stm-7 mutant allele GK-100 F11 containing a transfer DNA (T-DNA) insertion in the second intron of STM. Gray box represents UTR, black box represents exon, and line represents intron. (JPG 623 kb) [file 12915_2016_336_MOESM2_ESM.jpg]

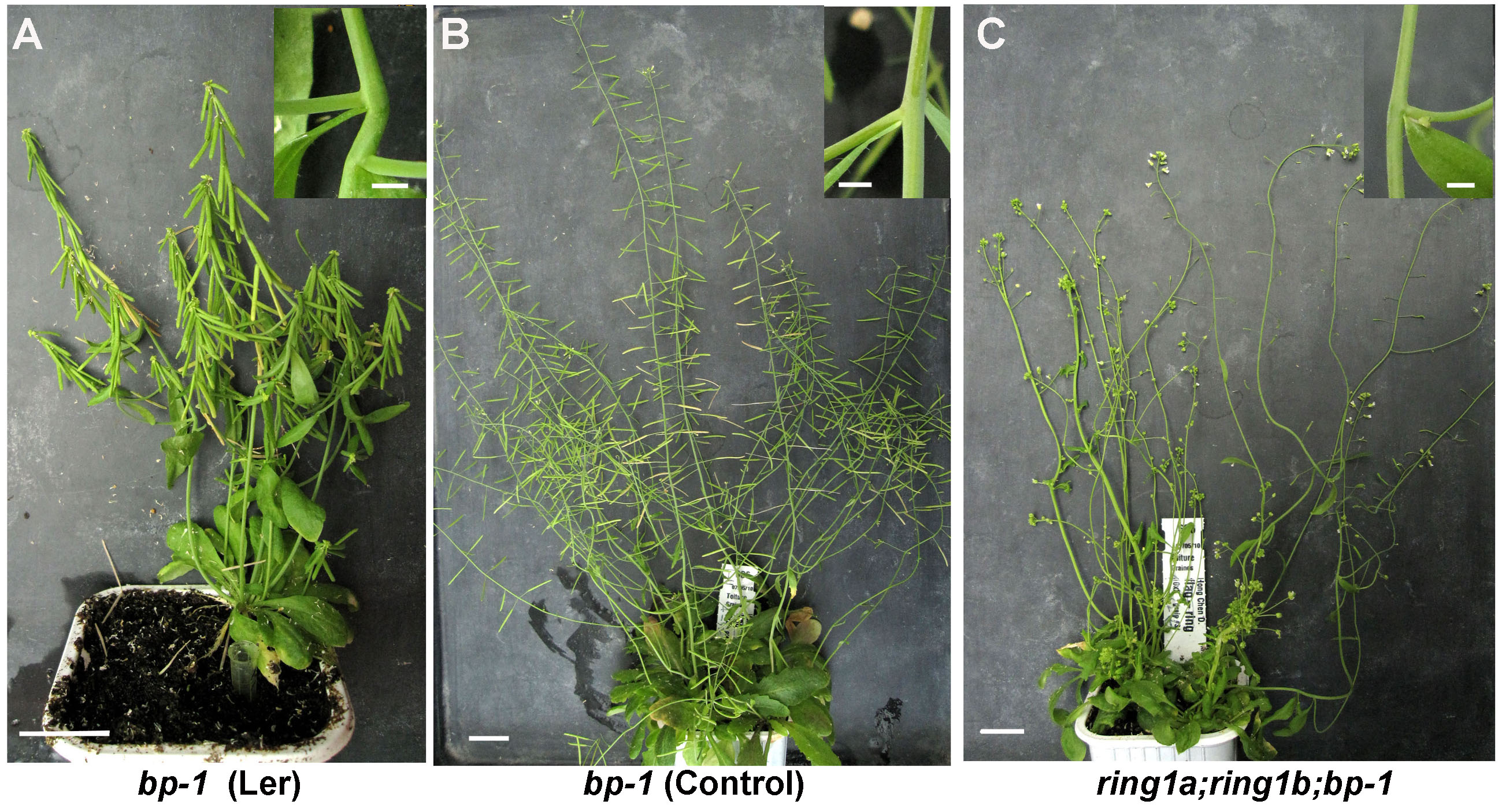

Supplement: Additional file 3: Figure S3. — Phenotype analysis of atring1a;atring1b;bp-1 plant. (A) Adult bp-1 plant (Ler). (B) Adult bp-1 control. (C) Adult atring1a;atring1b;bp-1 plant. The control bp-1 -/- mutant (B) and atring1a;atring1b;bp-1 triple mutant (C) are derived from the same F2 generation of atring1a -/+ ;atring1b -/+ ;bp-1 -/-. Insets indicate the close-up view of downward branch related to corresponding mutant. Bars = 2 cm in (A)–(C) and 1 mm in the corresponding insets. (JPG 711 kb) [file 12915_2016_336_MOESM3_ESM.jpg]

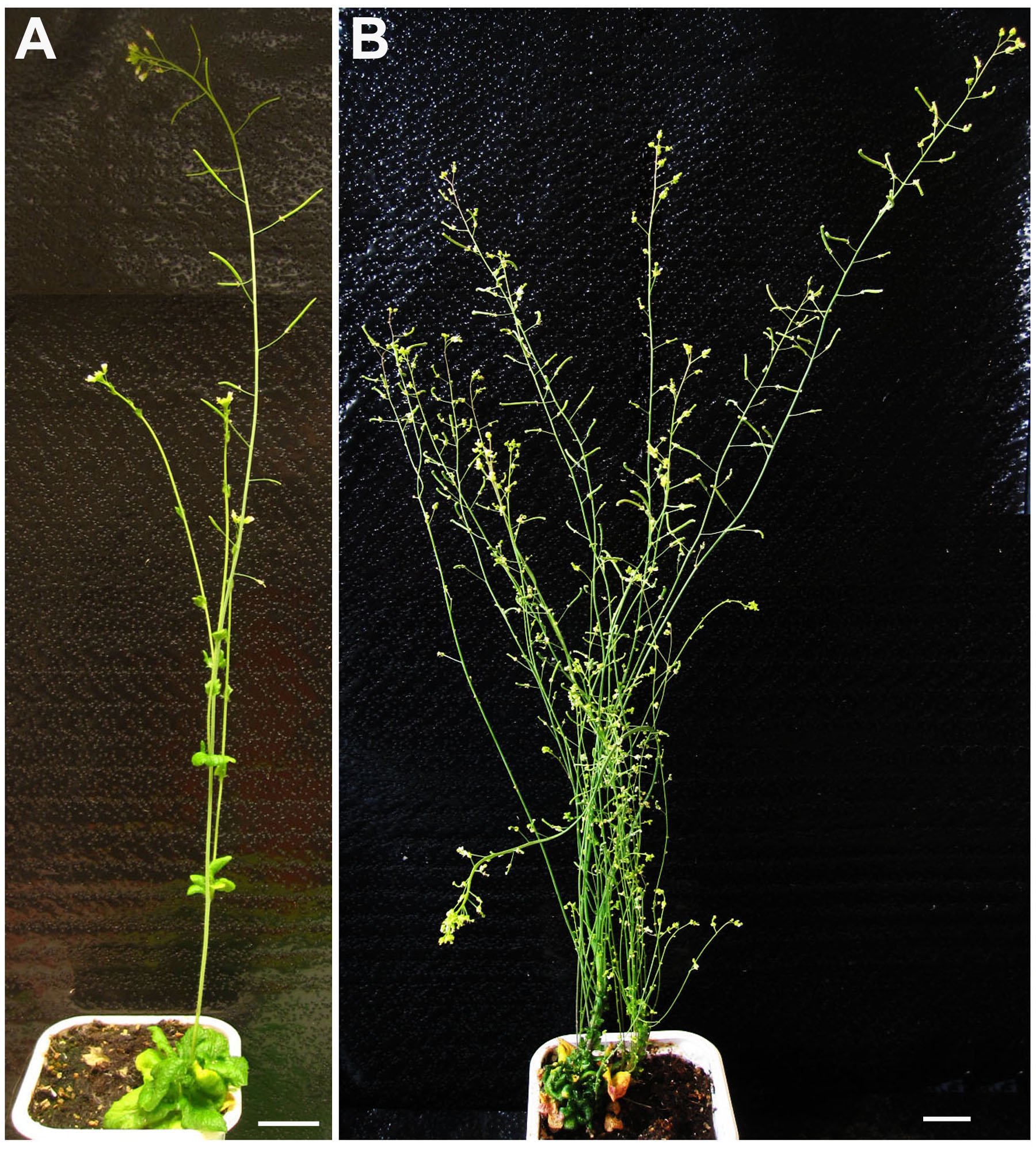

Supplement: Additional file 4: Figure S4. — Phenotype analysis of atring1a;atring1b;as1-1 mutant. (A) Adult plant architecture of as1-1 mutant. (B) Adult plant architecture of atring1a;atring1b;as1-1 mutant. Bars = 2 cm. (JPG 665 kb) [file 12915_2016_336_MOESM4_ESM.jpg]

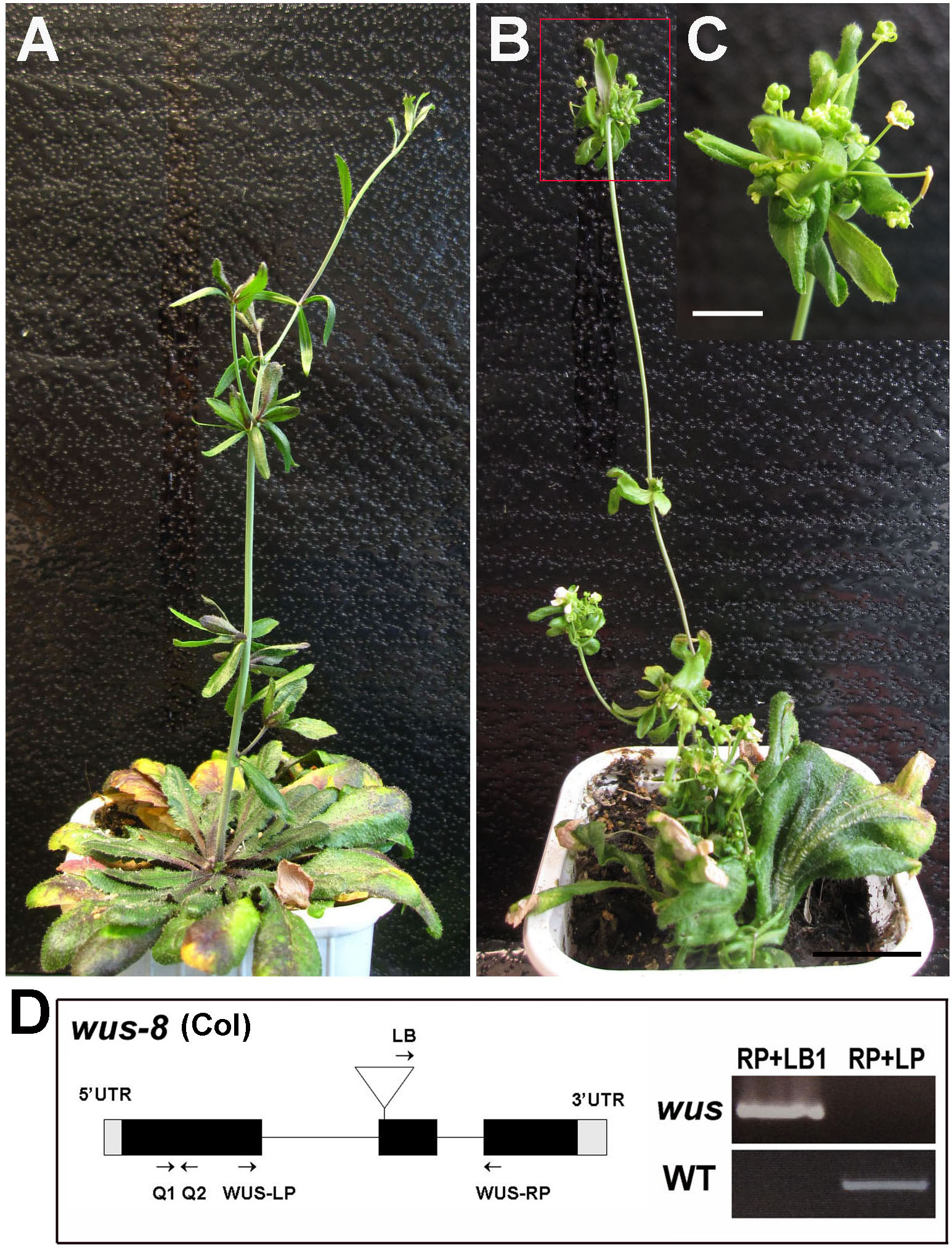

Supplement: Additional file 5: Figure S5. — Phenotype analysis of atring1a;atring1b;wus-8 triple mutant. (A) Adult plant of wus-8 mutant. (B) Adult plant of atring1a;atring1b;wus-8 triple mutant. (C) Close-up view of (B), showing the main florescence of atring1a;atring1b;wus-8 triple mutant. Bars = 2 cm in (A) and (B) and 500 μm in (C). (D) The wus-8 allele (SAIL_150_G06) harboring a T-DNA insertion in the second intron of WUS. Gray box represents UTR, black box represents exon, and line represents intron. (JPG 599 kb) [file 12915_2016_336_MOESM5_ESM.jpg]

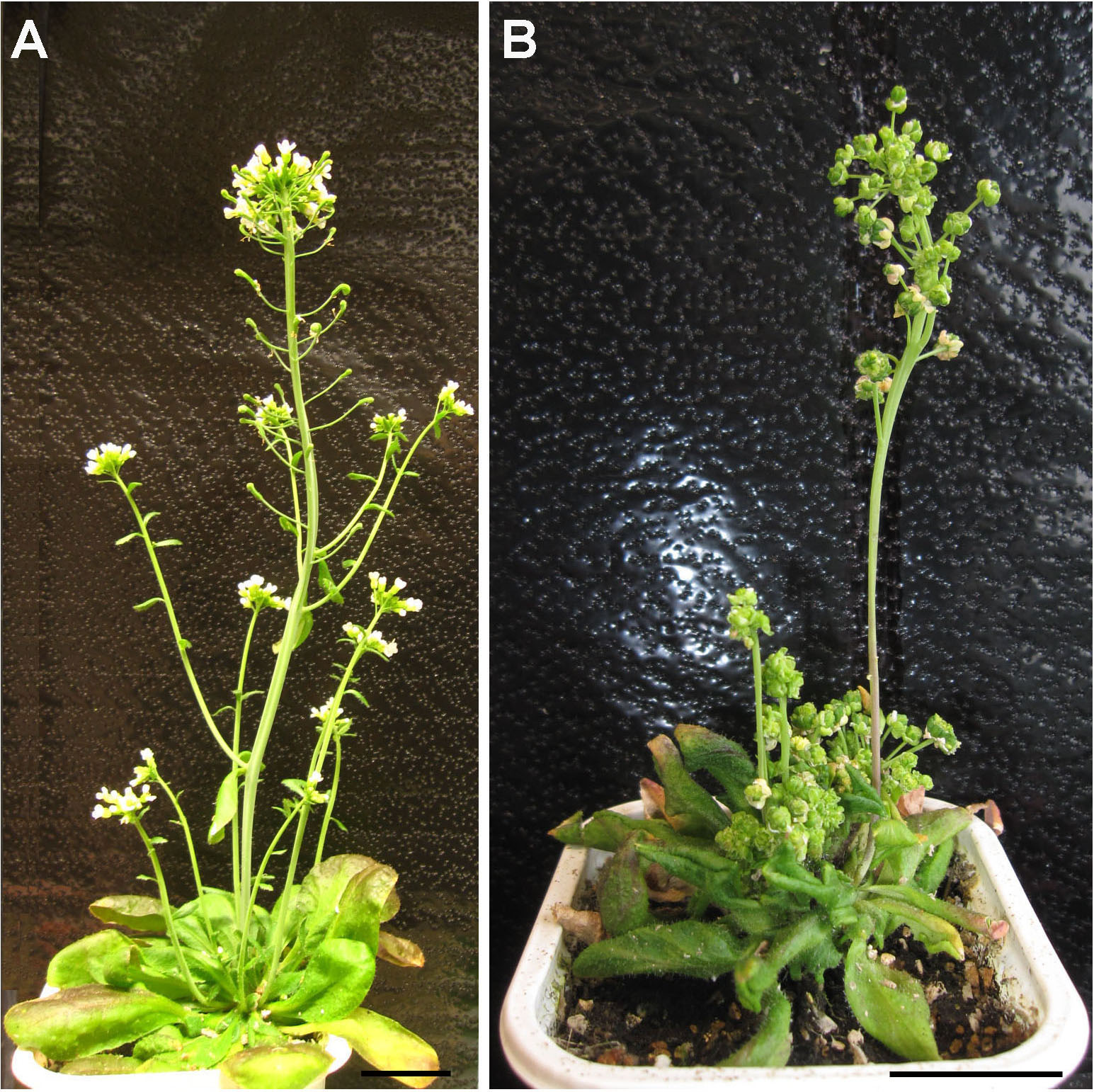

Supplement: Additional file 6: Figure S6. — Phenotype analysis of atring1a;atring1b;clv3-2 triple mutant. (A) Whole plant architecture of clv3-2 mutant. (B) Whole plant architecture of atring1a;atring1b;clv3-2 triple mutant. The control clv3-2 -/- mutant and atring1a;atring1b;clv3-2 triple mutant are derived from the same F2 generation of atring1a -/+ ;atring1b -/+ ;clv3-2 -/-. Bars = 2 cm. (JPG 521 kb) [file 12915_2016_336_MOESM6_ESM.jpg]
