# Supplementary material for: Arabidopsis PRC1 core component AtRING1 regulates stem cell-determining carpel development mainly through repression of class I KNOX genes
Source: BMC Biol. 2016 Dec 22;14:112. doi: 10.1186/s12915-016-0336-4 (PMC5178098; doi:10.1186/s12915-016-0336-4)
Supplement: Additional file 7: Table S1. — Primers used for genotyping, plasmid construction, and qRT-PCR. (DOC 86 kb) [file 12915_2016_336_MOESM7_ESM.doc]

| **Primers** | **Sequence (5'→3')** | **Note** |
| --- | --- | --- |
| **Genotyping** | | |
| RING1a-RP | TTCTATATCTGGAGACCAATC | AtRING1a |
| RING1a-LP | AGAACCTGTGGAGACATTCC |
| RING1a-RP | TTCTATATCTGGAGACCAATC | *ring1a*, AL_945948  Sulfadiazine resistance |
| O8409 | ATATTGACCATCATACTCATTGC |
| RING1b-RP | ATGCCTTCCTTGAAGAGCTTCTCC | AtRING1b |
| RING1b-LP | CAGATATCCGCTAGATCAACAACC |
| RING1b-RP | ATGCCTTCCTTGAAGAGCTTCTCC | *ring1b*, SALK_117985 |
| LBb1.3 | ATTTTGCCGATTTCGGAAC |
| STM-RP | TATCCTCACCTTCCTCTTTC | STM |
| STM-LP | CTATCAATTGCTGTCTCTCC |
| STM-RP | TATCCTCACCTTCCTCTTTC | *stm*, GK-100F11  Sulfadiazine resistance |
| O8409 | ATATTGACCATCATACTCATTGC |
| WUS-RP | GCTTTAATCCCGAGCGAC | WUS |
| WUS-LP | TAACAAGCCATATCCCAGC |
| WUS-RP | GCTTTAATCCCGAGCGAC | *wus*, SAIL_150_G06  Basta resistance |
| SAIL-LB1 | GCCTTTTCAGAAATGGATAAATAGCCTTGCTTCC |
| FIS2-RP | AAACCGAACCAGTTTTCATACC | FIS2 |
| FIS2-LP | TGTTGTTTCCATGATTTCTTTTTC |
| FIS2-RP | AAACCGAACCAGTTTTCATACC | *fis2*, SALK_009910 |
| LBb1.3 | ATTTTGCCGATTTCGGAAC |
| MET1-RP | GAT TGT GTC TCT ACT ACA GAG GC | MET1, AT5G49160 |
| MET1-LP | GTT AAG CTC ATT CAT AGC CTT GC |
| MET1-RP | GAT TGT GTC TCT ACT ACA GAG GC | *met1-3,* SAIL_809_E03  Basta resistance |
| MET1-BP | GGT TCT TAT AGG GTT TCG CTC |
| BMI1a-RP | CACATGCAGTTTTGGGGATAG | AtBMI1a |
| BMI1a-LP | TCTCTCCTTCCTCTTTCCTGG |
| BMI1a-RP | CACATGCAGTTTTGGGGATAG | *bmi1a*,WiscDsLox437G06  Basta resistance |
| p745 | AACGTCCGCAATGTGTTATTAAGTTGTC |
| BMI1b-RP | TGAAACCAGTAAAGCCGTGTC | AtBMI1b |
| BMI1b-LP | TTGTTGGTGGCGAACTTATTC |
| BMI1b-RP | TGAAACCAGTAAAGCCGTGTC | *bmi1b*, SALK_145041 |
| LBb1.3 | ATTTTGCCGATTTCGGAAC |
| GUS-LP | GTGGCAGTGAAGGGCGAACAGT | For GUS transgenic lines |
| GUS-RP | GTGAGCGTCGCAGAACATTACA |
| **Plasmid construction** | | |
| RING1b(-1586)F-*Sal*I | GCgtcgacGTACTGTTGAAGACGATTCATC | For  *RING1b::RING1b:GUS* |
| RING1b-R-*Bam*HI | TCggatccCGCGATTTGCTTTCTCCGGTACACT |
| BMI1c(-2126)F-*Sal*I | CGgtcgacGTTTGGACTCTTTTATTTCTGTTTCTGTTTTTG | For *BMI1c::BMI1c:GUS* |
| BMI1c-R-*Bam*HI | TCggatccTTCAGAGGCAGAGCCAGAGTCAGAGG |
| **qRT-PCR** | | |
| PP2A-Q1 | TAACGTGGCCAAAATGATGC | Internal reference |
| PP2A-Q2 | GTTCTCCACAACCGCTTGGT |
| RING1a-Q1 | ATCTCTGTTGCCGACCCACT |  |
| RING1a-Q2 | GCCGCATCTTCTCCTACTCT |
| RING1b-Q1 | TGAGAGGCAACGAAAAAAGC |  |
| RING1b-Q2 | AGTTCCACACAAGCACAGGT |
| BMI1a-Q1 | GGTCCCGTTTGGTTCTCACT |  |
| BMI1a-Q2 | TGTATTTCCATCCCTTATTCTC |
| BMI1b-Q1 | AGTTGTGTCCTCCATCTCATT |  |
| BMI1b-Q2 | GCTTTTGTTCTTTTTCCTGTT |
| BMI1c-Q1 | AACTTCACTGCGGGTCTCTTCA |  |
| BMI1c-Q2 | ACGGTCTCCCTATGTTTCTCCT |
| LFY-Q1 | TTGATGCTCTCTCCCAAGAAG |  |
| LFY-Q2 | TTGACCTGCGTCCCAGTAA |
| WUS-Q1 | CAGTTCGGAAAGATTGAGGG |  |
| WUS-Q2 | GGTGATGAAGATGGTGTGGT |
| AG-Q1 | TTATTCACTCCCGGCCATT |  |
| AG-Q2 | TGGCATAAGCTGCTCGTAGT |
| STM-Q1 | GCAACACATCCTCACCATTACTTCA |  |
| STM-Q2 | ATCAAAGCATGGTGGAGGAGA |
| BP-Q1 | TCCCATTCACATCCTCAACA |  |
| BP-Q2 | CCCCTCCGCTGTTATTCTCT |
| KNAT2-Q1 | AAACGCCATTGGAAGCCT |  |
| KNAT2-Q2 | ACAATGCACAATTTCATGTCTCTCT |
| KNAT6-Q1 | CCAAGAGAAGCAAGACAAGCTC |  |
| KNAT6-Q2 | CAGCTAATGCTATCTTATCTCCTTCAG |
| SHP1-Q1 | AAGCAGCAGTTAAGTGGTACTGAG |  |
| SHP1-Q2 | CCACCTTCCTCCATTGATCCTGAG |
| SHP2-Q1 | TCGTGTCCGATCCAAGAAGCAC |  |
| SHP2-Q2 | TCGATTCTTGTTGCTGTAGACCTG |
| STK-Q1 | GGGTGAAGCAAATTCTCAGG |  |
| STK-Q2 | CGATTTGTTGAGTTCTCTATCCTCT |
| **In situ hybridization** | | |
| AtRING1a-IS1 | GAGAAGATCTTTAGTGTGTGTCCC |  |
| AtRING1a-IS2 | AGAACCTGTGGAGACATTCC |  |
